# Itaconic acid underpins hepatocyte lipid metabolism in non-alcoholic fatty liver disease in male mice

**DOI:** 10.1038/s42255-023-00801-2

**Published:** 2023-06-12

**Authors:** Jonathan M. Weiss, Erika M. Palmieri, Marieli Gonzalez-Cotto, Ian A. Bettencourt, Emily L. Megill, Nathaniel W. Snyder, Daniel W. McVicar

**Affiliations:** 1 Cancer Innovation Laboratory, Center for Cancer Research, NCI Frederick, Frederick, MD USA; 2 Department of Cardiovascular Sciences, Lewis Katz School of Medicine at Temple University, Philadelphia, PA USA

**Keywords:** Metabolism, Metabolomics, Monocytes and macrophages, Fatty acids, Non-alcoholic fatty liver disease

## Abstract

Itaconate, the product of the decarboxylation of *cis*-aconitate, regulates numerous biological processes. We and others have revealed itaconate as a regulator of fatty acid β-oxidation, generation of mitochondrial reactive oxygen species and the metabolic interplay between resident macrophages and tumors. In the present study, we show that itaconic acid is upregulated in human non-alcoholic steatohepatitis and a mouse model of non-alcoholic fatty liver disease. Male mice deficient in the gene responsible for itaconate production (immunoresponsive gene (Irg)-1) have exacerbated lipid accumulation in the liver, glucose and insulin intolerance and mesenteric fat deposition. Treatment of mice with the itaconate derivative, 4-octyl itaconate, reverses dyslipidemia associated with high-fat diet feeding. Mechanistically, itaconate treatment of primary hepatocytes reduces lipid accumulation and increases their oxidative phosphorylation in a manner dependent upon fatty acid oxidation. We propose a model whereby macrophage-derived itaconate acts in *trans* upon hepatocytes to modulate the liver’s ability to metabolize fatty acids.

## Abstract (web summary)

In this study, Weiss, Palmieri et al. show that macrophage-derived itaconate impinges on hepatocyte lipid metabolism. This mechanism participates in the crosstalk of the immune system with hepatocytes during the development of non-alcoholic fatty liver disease.

## Main

Non-alcoholic fatty liver disease (NAFLD) is a global health crisis in adults and children^1^. Often associated with excess consumption of calories, accumulated adipose tissue and obesity, NAFLD represents a spectrum of liver disease, which is closely linked with inflammation, metabolic syndrome, insulin resistance and a host of risk factors for advanced disease such as liver cirrhosis and hepatocellular carcinoma^2–4^. As the predominant site for the uptake, storage and export of lipid, the liver plays an indispensable role in the metabolism of fat. The liver is a major site for the oxidation of triglycerides, generating fatty acids that are exported to the circulation, used by various tissues in the body for energy or stored in adipose tissue^3^. Hepatic lipid accumulation results from an imbalance between lipid availability and removal via fatty acid oxidation or lipoprotein secretion. Enhanced lipolysis in adipose tissue can result in higher circulating fatty acid levels and hepatic lipogenesis. When excess adiposity occurs, often due to dietary or genetic factors, free fatty acids released from adipose tissue may overwhelm the liver’s capacity to oxidize or export lipids, contributing to the development of fatty liver disease and steatosis. Obesity-associated changes in blood glucose levels and insulin intolerance can result in ‘lipid spillover’ characterized by alterations in lipid metabolism that contribute to high circulating levels of lipid and precede the development of metabolic syndromes such as type 2 diabetes, atherosclerosis and a progression of hepatic steatosis, fatty liver disease and liver cirrhosis^3,4^. A better understanding of the metabolic regulation of lipid metabolism by the liver may facilitate the development of therapeutic interventions that counteract this growing health crisis.

Regulation of lipid homeostasis and metabolism is complex, governed by a large array of hormones, metabolites and transcription factors^2,3^. Among metabolites, acetyl-coenzyme A (acetyl-CoA), a product of the oxidation of pyruvate during glycolysis, β-oxidation of fatty acids or metabolism of certain amino acids, plays a central role in lipid metabolism^3^. In addition to its role as the entry point into the citric acid (TCA) cycle, acetyl-CoA also serves as a principal precursor for the synthesis of fatty acids and lipids. We hypothesized that other metabolic intermediates might exert as yet poorly defined roles in lipid metabolism. As one example, we and others showed previously that the immunometabolite itaconic acid^5^ (itaconate) regulated β-oxidation of lipids promoting mitochondrial reactive oxygen species (ROS) and oxidative phosphorylation of macrophages^6,7^. Itaconate is formed by diverting *cis*-aconitate away from the TCA during inflammatory macrophage activation, a decarboxylation reaction catalyzed by the enzyme aconitate decarboxylase 1 (*Acod1*, referred to here as immunoresponsive gene-1; *Irg1*)^8^. Itaconate has been described as an anti-inflammatory immunometabolite that exerts regulatory roles in numerous pathologies, including cancer, infection, sepsis and tissue injury responses^7,9–13^. Metabolism of itaconate begins with its activation to itaconyl-CoA by succinyl-CoA ligase, which has been postulated to serve as a ‘CoA sink’ that may regulate fatty acid metabolism and other CoA-dependent pathways^14^.

In the present study, we identify an important and underappreciated role for itaconic acid in lipid metabolism in the liver and the progression of NAFLD. We find that both *Irg1* and itaconate accumulates in liver macrophages in a mouse model of NAFLD and human NASH livers. Mice with global or myeloid-specific deletion of Irg1 demonstrated a dramatic accumulation in adiposity, exacerbated lipid accumulation (prominently triglycerides) in the liver and exacerbated glucose and insulin intolerance. Mechanistically, we find that macrophage-derived itaconate acts *in trans* to alter the oxidative profile and lipid metabolism of hepatocytes. Our results reveal underappreciated pathways whereby itaconate regulates hepatocyte responses to lipid and may help identify *Irg1* and itaconate as potential targets during NAFLD.

## Results

### Lipid metabolism in macrophages lacking Irg1 is dysregulated

**Fig. 1:**
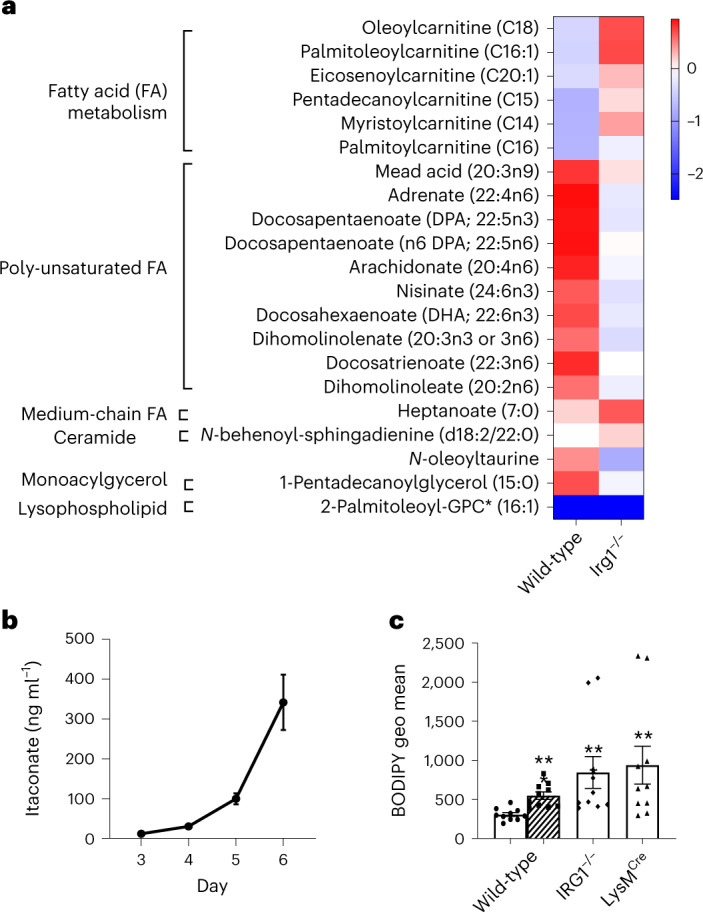
Dysregulated lipid metabolism in *Irg1*^−/−^ macrophages. **a**, BMMs from four wild-type and four Irg1^−/−^ mice were analyzed for broad metabolomics by mass spectrometry. For each metabolite, a ratio was computed by dividing the metabolite level by the relative mean for all eight samples (four wild-type and four *Irg1*^−/−^). The heat map depicts log_2_-transformed ratios for metabolites significantly different; all metabolites shown are *P < 0.05 between genotypes, as determined by two-sided multiple *t*-tests (one per row). **b**, Itaconate production was confirmed in cultured BMMs by culturing cells in the presence of 50 μg ml^−1^ macrophage colony-stimulating factor. At the indicated times, supernatant was collected and analyzed for itaconate using mass spectrometry. The graph depicts results from triplicate bone-marrow cultures. **c**, Peritoneal resident macrophages were isolated from the indicated mice. Lipid staining was visualized using flow cytometric analysis of BODIPY 493/503 staining. The graph illustrates results from ten mice. Data are presented as mean ± s.e.m. A two-sided analysis of variance (ANOVA) was performed between each group and the wild-type control (**P = 0.0015; ***P = 0.0001). As a positive control, wild-type mice were treated with 50 μg LPS 24 h before the isolation of peritoneal macrophages. Source data

A role for the immunometabolite itaconic acid in the β-oxidation of lipids as a source of fuel for oxidative phosphorylation and mitochondrial ROS has been reported in tumor-associated macrophages, J774.2 and zebrafish macrophage lineage cells^6,7^. We asked whether *Irg*1 might regulate lipid homeostasis in primary bone-marrow macrophages (BMMs). Using unbiased metabolomics, we found that *Irg1*^−/−^ mice have dysregulated lipid metabolism compared to wild-type cells. BMMs from *Irg1*^−/−^ mice showed dramatic accumulation of carnitine conjugated fatty acids, along with reduced levels of many long-chain polyunsaturated fatty acids (Fig. 1a). Although the BMMs in this analysis were unstimulated, we confirmed itaconate production during BMM culture using mass spectrometry (Fig. 1b) suggesting that exposure of BMMs to itaconate during derivation affects their lipid metabolism.

We also detected a similar lipid dysfunction in resident peritoneal macrophages. Unstimulated peritoneal resident macrophages from *Irg1*^−/−^ mice as well as *Irg1*^fl/fl^ × LysM^Cre^ mice had significantly elevated neutral lipid content as detected by BODIPY staining compared to cells from wild-type mice (Fig. 1c). Taken together, these results prompted us to investigate the role for *Irg1* and itaconic acid in a mouse model of hyperlipidemia.

### Irg1 expression and itaconate production are associated with dyslipidemia in mice fed western diet and human NASH

**Fig. 2:**
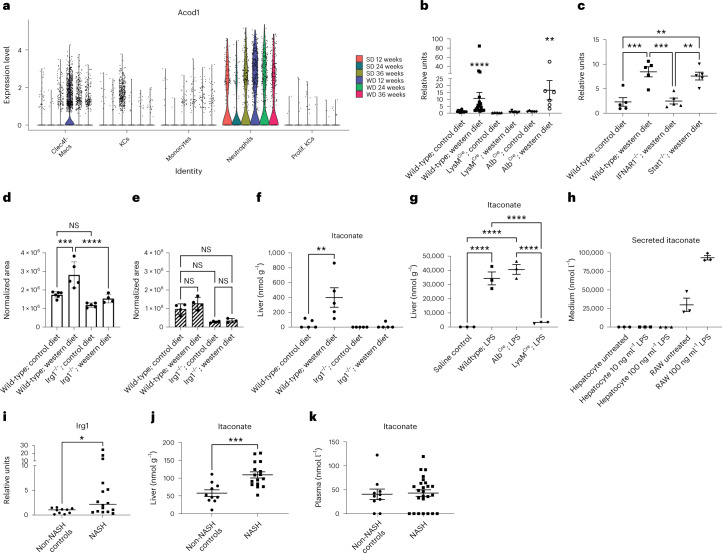
Irg1 is upregulated in hepatic macrophages in response to high-fat diet. **a**, Transcriptomic analysis of *Acod1/Irg1* mRNA expression on cell subsets in mice fed control/standard diet (SD) or WD for the indicated times from a single-cell RNA-seq experiment in Guilliams et al.^17^ **b**,**c**, Whole liver lobes were collected on week 12 following control or WD feeding of the indicated mice. Murine *Irg1* gene expression was quantified in each sample using qPCR. For **b**, *n* = 20 wild-type mice per group; *n* = 6 Cre-specific mice per group (****P < 0.0001; **P = 0.002). For **c**, *n* = 5 mice per group (**P = 0.003; ***P = 0.0005). **d**,**e**, F4/80^+^ macrophages were isolated by magnetic selection from single-cell suspensions of liver cells. Graphs depict the relative area of itaconate production in positively selected F4/80^+^ macrophages (***P = 0.0002; ****P < 0.0001; NS, not significant) (**d**) and the flow-through non-macrophage fraction (*n* = 5 mice per group) (**e**). **f**, Itaconate production was quantified from homogenized livers of 12 week WD-fed mice, normalized for liver tissue weight (*n* = 5 mice per group; *P* = 0.008). **g**, Itaconate production was quantified from mice treated with 50 μg LPS overnight (*n* = 3 mice per group; *P* < 0.0001). **h**, Secreted itaconate in the supernatants of untreated and LPS-treated hepatocytes or RAW 264.7 cells as a positive control (*n* = 3 per group). A two-sided ANOVA with multiple comparisons was used for statistical analyses (**b**–**g**). **i**,**j**, Human *Irg1* expression (*P = 0.03) (**i**) and itaconate production (**j**) (***P = 0.017) in human NASH livers. Human liver samples from 10 non-NASH controls (consisting of hemangioma, focal nodular hyperplasia, hepatic adenoma, hepatocellular carcinoma and benign cases) and 16 NASH cases were analyzed for itaconate expression using mass spectrometry. Results for *Irg1* are shown as the relative fold change over non-NASH controls. Results for itaconate were normalized per mg liver tissue. A two-sided Mann–Whitney *U*-test was used for statistical analysis of human NASH samples (**i**,**j**). **k**, Itaconate levels in the plasma of non-NASH controls and NASH cases. Data are presented as mean ± s.e.m. Source data

Mice fed a western diet (WD) for 12 weeks develop hyperlipidemia in the liver, denoted by extensive lipid accumulation, elevated serum triglycerides and insulin resistance^15,16^. Our analysis of previously published transcriptomic data of hepatic myeloid cells in WD-fed wild-type mice^17^ indicated *Irg1* expression was upregulated in the Clec4f^−^ macrophage population, which is derived from immigration of circulating macrophages following 12–36 weeks of WD feeding (Fig. 2a). *Irg1* expression was detectable in resident Kupffer cells (KCs); however, this expression dramatically declined with WD feeding and no *Irg1* expression was detectable in proliferating KCs. Robust *Irg*1 expression was noted in neutrophils; however, this was completely independent of WD feeding, suggesting that the macrophage but not neutrophil expression of *Irg1* was related to hyperlipidemia. Because *Irg1* expression has been reported in hepatocytes during ischemia-reperfusion disease^10^, we investigated further the cellular source of hepatic *Irg1* and itaconic acid and interrogated the role of *Irg1*-catalyzed itaconic acid during WD feeding. We compared lipid pathologies between wild-type and *Irg1*^−/−^ mice bred onto identical genetic backgrounds. Quantitative PCR analysis on livers confirmed induction of *Irg1* mRNA after 12 weeks of WD feeding (Fig. 2b) but not after 9 weeks of WD feeding (Supplementary Fig. 1a). Molecular analysis of livers from mice bearing tissue-specific deletion of *Irg1* showed that its expression was confined to the myeloid compartment as WD-induced *Irg1* expression was abrogated in *Irg1*^fl/fl^ × LysM^Cre^ but preserved in *Irg1*^*f*l/fl^ × Alb^Cre^ mice that have the gene deleted in hepatocytes (Fig. 2b). No *Irg1* expression was detected in macrophages isolated from adipose tissue. Further interrogation indicated that *Irg1* upregulation in the liver of WD-fed mice was dependent on interferon receptor (IFNAR) expression but independent of Stat1 expression (Fig. 2c), implicating non-canonical type I interferon signaling or potentially Stat2 homodimers as being important to the upregulated Irg1 expression in the liver following WD^18^. Mass spectrometry confirmed the accumulation of itaconic acid in isolated F4/80-positive macrophages from these livers (Fig. 2d), whereas no itaconic acid was expressed in F4/80-negative cell fractions (Fig. 2e). We next identified the pathophysiological concentration of itaconate in the liver and blood of WD-fed mice. Approximately 400 ± 131 μM itaconate was detected in the livers of WD-fed mice, which was significantly increased compared to control diet-fed mice (Fig. 2f). In contrast, itaconate was not increased in the blood after WD feeding (Supplementary Fig. 1b). Lipopolysaccharide (LPS) treatment of BMMs from wild-type and *Irg1*^−/−^ mice confirmed the dependence of itaconate production on *Irg1* expression (Supplementary Fig. 1c,d). To further confirm that hepatocytes are not a significant source of itaconate in our studies, we treated mice with LPS and found that itaconate levels in Irg1^fl/fl^ × Alb^Cre^ mice were indistinguishable from wild-type mice (Fig. 2g). Further, LPS-exposed hepatocytes did not produce detectable itaconate (Fig. 2h). Together these data demonstrate that loss of *Irg1* is associated with lipid accumulation in macrophages and that WD feeding results in *Irg1* expression and itaconate production in hepatic macrophages, but not hepatocytes. To determine whether itaconate was upregulated in human non-alcoholic steatohepatitis (NASH), we obtained liver biopsies from 10 non-NASH controls and 16 patients with NASH. By qPCR, *Irg1* messenger RNA expression was significantly increased in NASH compared to non-NASH controls (Fig. 2i). Furthermore, itaconate levels were significantly higher in liver tissue from human NASH (approximately 109 ± 9 μM) compared to non-NASH controls (Fig. 2j) but again itaconate levels in the blood were unaltered between control and patients with NASH (Fig. 2k). The diagnosis of NASH provided to us by the University of Pittsburgh was further corroborated by a significant upregulation of interleukin-6 mRNA expression in liver NASH samples (Supplementary Fig. 2a). Among other metabolites, we found succinate and glutamine were unchanged, whereas lactate, citrate and α-ketoglutarate were significantly upregulated in NASH (Supplementary Fig. 2b–f).

**Fig. 3:**
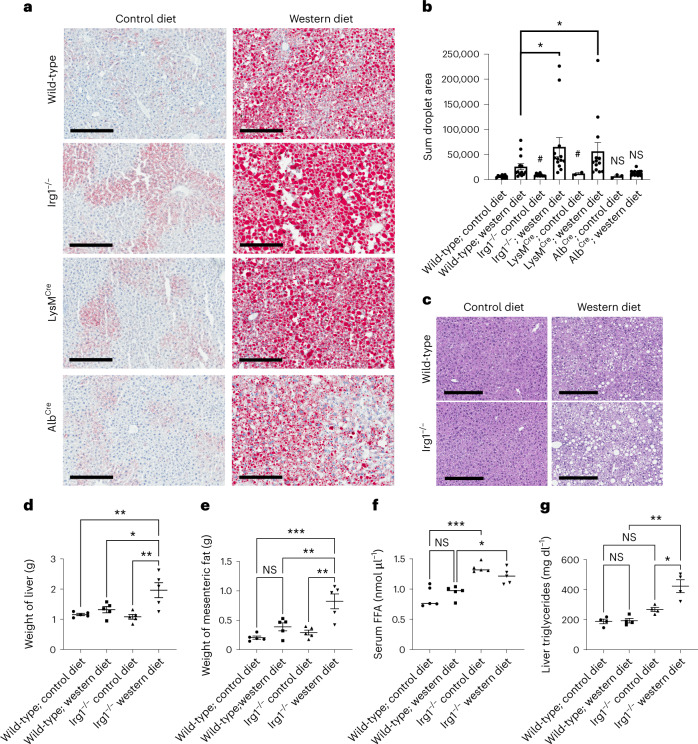
Exacerbated fatty liver disease in *Irg1*^−/−^ mice after WD diet feeding. **a**, ORO staining of frozen liver tissue sections. Each image is representative of ten non-overlapping fields from five mice per group and repeated in two separate experiments; scale bar, 300 μm). **b**, The sum areas of ORO staining were quantified using ImageJ software (*P = 0.02 for the indicated comparisons; #*P* = 0.01 compared to the wild-type/control diet sample; *n* = 10). **c**, Representative H&E staining of liver tissues. The image depicted is representative of five non-overlapping fields from three mice. Scale bar, 300 μM; ×10 magnification. **d**, Liver weights of five mice per group (*P = 0.036; **P = 0.009). **e**, Mesenteric fat weights (five mice per group; **P = 0.009; ***P = 0.0006; NS, not significant). **f**, Serum levels of free fatty acids (*n* = 5 per group; *P = 0.043; ***P = 0.0006; NS, not significant). **g**, Liver triglycerides (*n* = 4 per group; *P = 0.013; **P = 0.001; NS, not significant). Data are presented as mean ± s.e.m. A two-sided ANOVA with multiple comparisons was used for statistical analyses. Source data

Notably, *Irg1*^−/−^ and *Irg1*^fl/fl^ × *LysM*^Cre^ mice fed WD exhibited increased lipid accumulation in the liver compared to wild-type mice (Fig. 3a,b for Oil Red O (ORO) staining and Fig. 3c for hematoxylin and eosin (H&E) staining). In contrast, *Irg1*^fl/fl^ × *Alb*^Cre^ mice fed a WD did not exhibit any exacerbation in diet-induced lipid accumulation. Notably, *Irg1*^−/−^ mice fed control chow diet also had significantly increased lipid staining compared to control wild-type mice, suggesting an important role for *Irg1* in both homeostatic and pathogenic lipid metabolism in the liver. Adipose and liver tissue from WD-fed mice but not control diet-fed mice was significantly increased in weight (Fig. 3d,e) and *Irg1*^−/−^ mice fed either control or WDs had significantly elevated free fatty acids in the blood (Fig. 3f) and increases in liver triglycerides (Fig. 3g). No differences in serum triglycerides or cholesterol were observed nor was there any difference in adipose triglycerides (Supplementary Fig. 3).

**Fig. 4:**
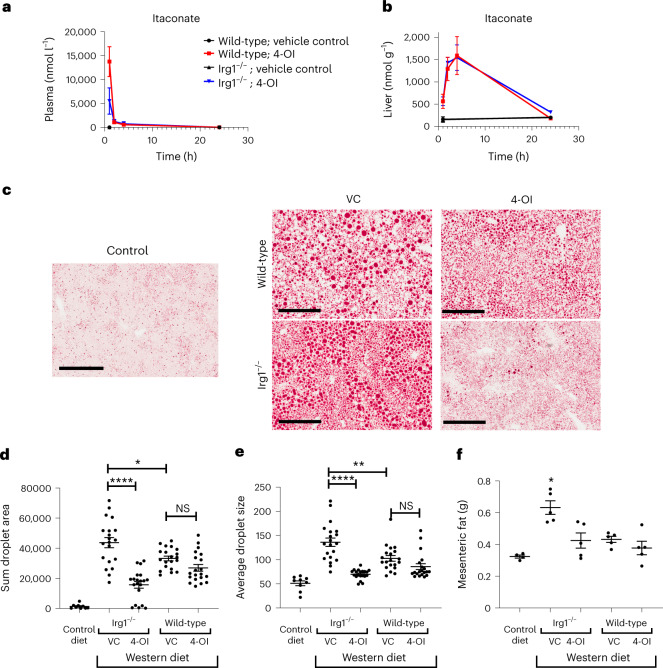
Treatment of mice with 4-OI reverses the exacerbated fatty liver disease in *Irg1*^−/−^ mice. Mice on WD were treated with 50 mg kg^−1^ 4-OI in 40% cyclodextrin/PBS or vehicle control (40% cyclodextrin/PBS) for 12 weeks. **a**,**b**, Itaconate levels in the plasma (**a**) and liver (**b**) were quantified at the indicated times (*n* = 5 mice per group). **c**, ORO staining of control diet- (top) and WD- (bottom) fed mice. The images are each representative of 20 non-overlapping images from five different mice per group. **d**, The sum areas of ORO staining were quantified using ImageJ software (*P = 0.015; ****P < 0.0001 for the indicated comparisons; *n* = 20). **e**, Quantitation of ORO (average droplet size) was obtained by dividing the sums of droplet area (**d**) by the number of droplets/image (**P = 0.002; ****P < 0.0001; *n* = 20). **f**, Mesenteric fat weight (five mice per group; *P* = 0.014 for the indicated group, as compared to all other groups). Data are presented as mean ± s.e.m. A two-sided ANOVA with multiple comparisons was used for statistical analyses. Source data

Having established an important role for itaconate in the regulation of lipid metabolism in the liver, we asked whether treatment of WD-fed mice with the cell-permeable derivative 4-octyl itaconate (4-OI) could ameliorate dysfunctional lipid metabolism in *Irg1*^−/−^ mice. We assayed the kinetics of blood and liver itaconate levels following a single administration of 4-OI. The administration of a single dose of 4-OI resulted in transient high levels of itaconate in the blood (Fig. 4a), which fell dramatically in the first few hours. In contrast, itaconate levels in the liver peaked at 4 h after administration, falling back to baseline within 24 h (Fig. 4b). 4-OI treatment significantly reduced liver ORO staining (Fig. 4c–e) and mesenteric fat weight (Fig. 4f). These findings indicate that exogenous administration of near-physiologically relevant levels of itaconate can successfully reverse the severe hyperlipidemic phenotype in WD-fed *Irg1*^−/−^ mice.

**Fig. 5:**
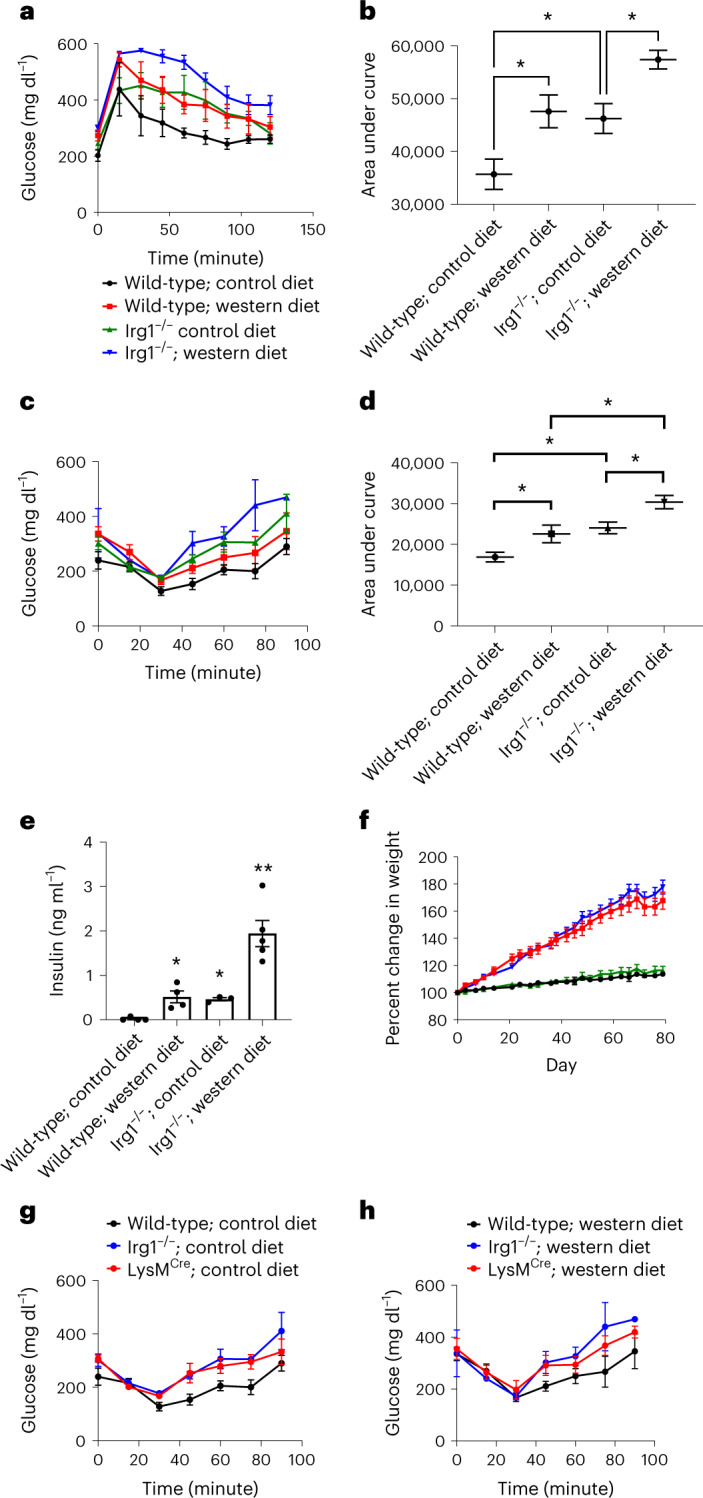
Glucose and insulin intolerance in *Irg1*^−/−^ and *Irg1*^fl/fl^ × *LysM*^Cre^ mice after WD feeding. **a**, Blood glucose levels following a single dose challenge of 2 g kg^−1^ glucose of the indicated mice. The graph depicts *n* = 5 mice from one experiment, representative of three total experiments. **b**, Area under the curve for the glucose tolerance test (*P = 0.04; *n* = 5). **c**, Blood glucose levels following a single dose challenge of 0.5 U kg^−1^ insulin of the indicated mice (*n* = 5 mice per group). **d**, Area under the curve for the insulin tolerance test (*P = 0.031; *n* = 5). **e**, Serum insulin levels at 12 weeks (*P = 0.03; **P = 0.001; *n* = 5 mice per group). **f**, Mouse weight measurements, as percentage of day 0 starting weight, during the 12 weeks of control or WD feeding. **g**,**h**, Insulin intolerance in *Irg1*^fl/fl^ × *LysM*^Cre^ mice was determined by feeding mice control (**g**) or WD (**h**) for 12 weeks and blood glucose levels were monitored over time following a single-dose challenge of insulin of the indicated mice. The graphs depict *n* = 5 mice per experiment, representative of three similar experiments. Data are presented as mean ± s.e.m. A two-sided ANOVA with multiple comparisons was used for statistical analyses. Source data

Given the changes in liver lipid burden we assessed characteristics of metabolic syndrome in mice lacking Irg1. *Irg1*^−/−^ mice fed either control or WD develop glucose intolerance as compared to respective diet-fed wild-type mice (Fig. 5a,b). *Irg1*^−/−^ mice on WD also had insulin resistance (Fig. 5c,d) as well as significantly elevated blood insulin levels (Fig. 5e). These findings were evident even though no differences were observed in body weight between wild-type and *Irg*1^−/−^ mice (Fig. 5f) and no differences were observed in the amount of food intake by the mice (Supplementary Fig. 4). Consistent with the patterns of expression we documented, *Irg*1 deficiency in the myeloid compartment was sufficient to yield elevated insulin resistance as *Irg*1^fl/fl^ × *LysM*^Cre^ mice exhibited similar insulin intolerance as global *Irg1*^−/−^ mice; regardless of whether mice were fed a control diet (Fig. 5g) or WD (Fig. 5h).

**Fig. 6:**
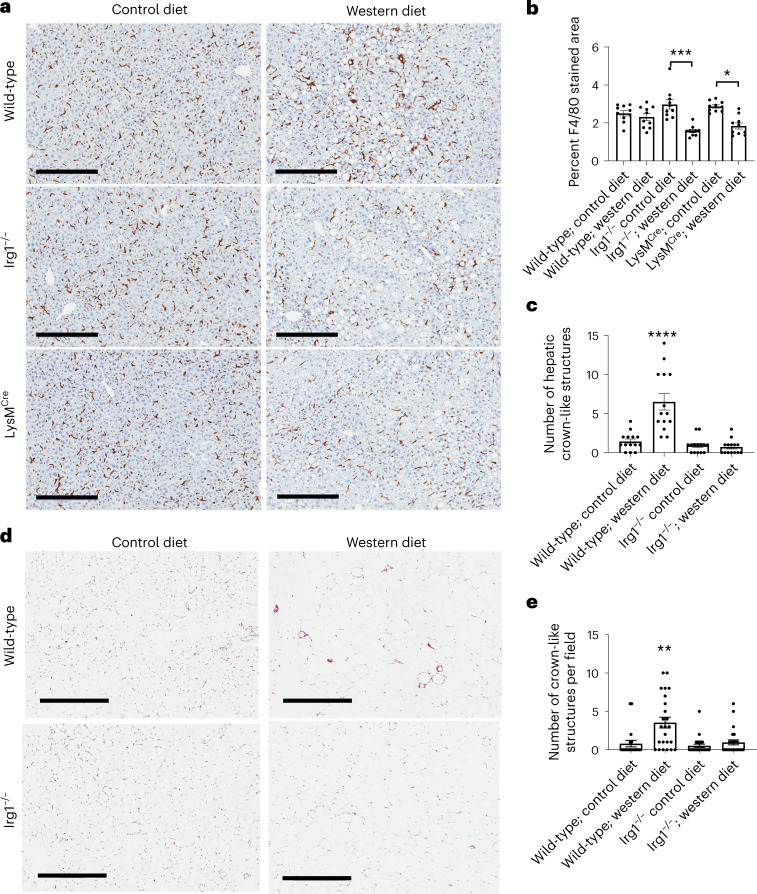
Loss of hepatic and adipose macrophages in global and myeloid-deficient Irg1 mice fed WD. **a**, Macrophage (F4/80) staining of formalin-fixed liver tissues, visualized using DAB chromogen. Each image is representative of ten non-overlapping fields from five mice per group and repeated in two separate experiments; scale bar, 300 μm). **b**, The F4/80-stained areas of 14 images per group were quantitated using ImageJ software (*P = 0.015; ***P = 0.0002). **c**, Reduction of hepatic crown-like structures (CLSs); F4/80^+^ macrophages forming a ring-like structure. The number of CLSs were manually counted on 14 images per group (****P < 0.0001). **d**, Macrophage (F4/80) staining of formalin-fixed adipose tissue, visualized using alkaline phosphatase chromogen (scale bar, 500 μM). Each image is representative of five non-overlapping fields from five mice per group. **e**, Quantitation of CLSs in adipose tissue (*n* = 25 images; **P = 0.002). CLSs in adipose tissue surround dying adipocytes in a crown-like pattern. Data are presented as mean ± s.e.m. A two-sided ANOVA with multiple comparisons was used for statistical analyses. Source data

WD-driven metabolic disorder is associated with substantial remodeling of adipose tissue by lipid-associated macrophages (LAMs). Even though we did not detect *Irg1* expression in LAMs during control or WD feeding, the increased mesenteric fat burden of *Irg1*^−/−^ mice prompted us to assess LAM number and architecture. We found the accumulation of lipid in the mesenteric fat and liver in mice lacking *Irg1* was accompanied by a notable loss of F4/80^+^ macrophages in both these tissues. In the liver, significant reductions in the frequencies of F4/80^+^ cells were observed in both *Irg*1^−/−^ and *Irg*1^fl/fl^ × *LysM*^Cre^ mice fed a WD (Fig. 6a,b) and hepatic crown-like structures were severely reduced (Fig. 6c). Similarly, F4/80^+^ cells were reduced, and crown-like structures were nearly absent in adipose tissue of *Irg*1^−/−^ mice fed a WD (Fig. 6d,e). Given the important homeostatic role for hepatic and adipose macrophages, our findings suggest that their notable loss in *Irg1*^−/−^ mice accompanies dysregulated lipid metabolism.

### Itaconate regulates lipid metabolism by enhancing fatty acid oxidation in hepatocytes

**Fig. 7:**
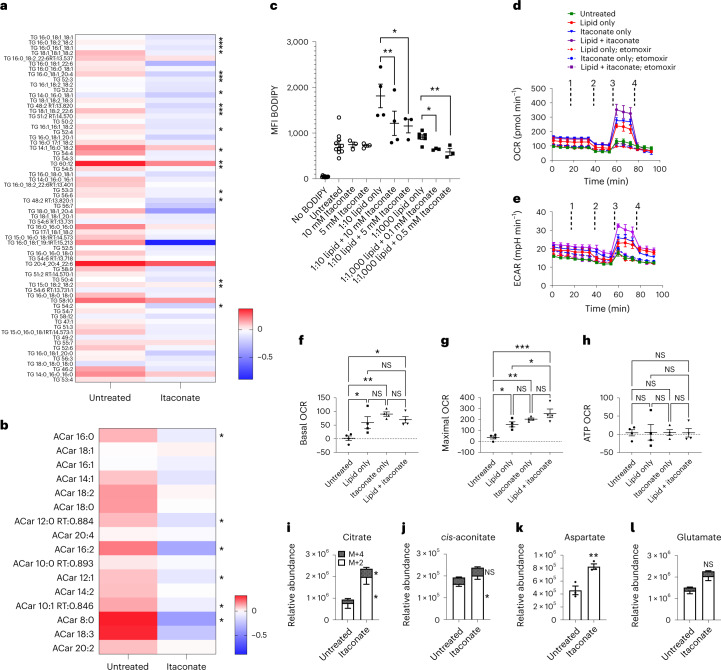
Itaconate reduces lipid accumulation by hepatocytes and enhances their oxidative phosphorylation. Targeted lipidomics showing triglycerides (**a**) and acyl carnitines (**b**) between untreated and itaconate-treated hepatocytes. For each metabolite, a ratio was computed by dividing the metabolite level by the relative mean for all six samples (three wild-type and three *Irg1*^−/−^). The heat map depicts log_2_-transformed ratios for metabolites. Significance was determined by two-sided multiple *t*-tests (one per row between genotypes) and is denoted on the side of the heat maps. **c**, Primary cultures of hepatocytes were treated with the indicated doses of itaconate and/or the indicated dilution of chemically defined lipid mixture. After 24 h, the treated hepatocytes were collected and analyzed for BODIPY staining. The graph depicts hepatocytes treated with itaconate and/or lipid (*n* = 3 from one experiment shown representative of seven similar experiments; *P = 0.028; **P = 0.005). **d**,**e**, Hepatocytes seeded in 96-well plates were treated with 10 mM itaconate and/or 1:100 lipid. The next day, cells were washed and analyzed using a Seahorse Bioanalyzer. **d**,**e**, The OCR (**d**) and extracellular acidification rate (ECAR) (**e**) is shown for one experiment (four replicate wells). Similar results were obtained in five similar experiments. At the indicated times, drugs were injected as (1) medium only or 40 μM etomoxir; (2) 1 μg ml^−1^ oligomycin; (3) 1 μM FCCP; (4) 100 nM rotenone + 1 μM antimycin A. **f**–**h**, The basal (**f**), maximal (**g**) and ATP OCR (**h**) are graphed (*n* = 4 for each; *P < 0.05; **P = 0.003). A two-sided ANOVA with multiple comparisons was used for statistical analyses in **c**,**f**–**h**. **i**–**l**, For detection of fatty acid carbon utilization into TCA-cycle intermediates hepatocytes were cultured overnight in 1:10 lipid mix ± 10 mM itaconate. Uniformly (U-13C16) labeled palmitate was added for the final 4 h and intracellular levels of labeled TCA intermediates were measured by mass spectrometry. Open bars depict the mean ± s.e.m. for M + 2 isotopologs and dark bars depict the mean ± s.e.m. of M + 4 isotopologs (*P = 0.04; **P = 0.006). No M + 4 was detected for aspartate. Statistical differences for (**i**–**l**) were determined by two sample *t*-test (*n* = 3 per group). Source data

To begin to reveal the mechanisms behind these phenomena, we isolated F4/80^+^ macrophages from the livers of wild-type or *Irg1*^−/−^ mice and performed targeted metabolomics. These data confirmed preferential accumulation of several long-chain fatty acids in the macrophages from *Irg1*^−/−^ mice fed a WD (Supplementary Fig. 5a). Moreover, several key metabolites associated with mitochondrial function, including pyruvate, fumarate, citrate and oxaloacetate were reduced in macrophages from WD-fed *Irg1*^−/−^ mice compared to their wild-type counterparts (Supplementary Fig. 5b). These data suggest subdued mitochondrial function in the absence of *Irg*1. We considered that metabolic dysfunction in hepatic macrophages alone would be unlikely to fully explain the substantial liver disease phenotype of mice lacking *Irg*1, but that these cells may represent sentinels of greater liver metabolic changes. Based on the lipid dysfunction we observed in BMMs and liver macrophages lacking *Irg*1, we hypothesized that myeloid-derived itaconic acid might act in *trans* to modulate lipid metabolism by hepatocytes. We tested this possibility by assessing the metabolic response of hepatocytes to exogenous itaconate. Exogenous itaconate readily accumulated in hepatocytes (Supplementary Fig. 6a). Itaconate was not sufficient to cause succinate accumulation (Supplementary Fig. 6b) in hepatocytes; nor did itaconate cause alterations in *cis*-aconitate or pyruvate levels (Supplementary Fig. 6c,d, respectively). Next, we assayed lipid accumulation in cultures of primary hepatocytes challenged with exogenous lipid with or without ex vivo treatment with sodium itaconate^19^. Targeted lipidomics indicated that multiple triglycerides (Fig. 7a) and acyl carnitines (Fig. 7b) were significantly downregulated in itaconate-treated hepatocytes. Moreover, hepatocytes challenged with lipid showed the expected increase in BODIPY staining and exposure to doses of physiologically relevant concentrations of itaconate ranging as low as 0.1 mM reduced that lipid staining (Fig. 7c). By both BODIPY staining of neutral lipids (Fig. 7c) and targeted lipidomics (Supplementary Fig. 7a), significantly reduced lipid accumulation was observed in itaconate-treated hepatocytes. Similarly, itaconate treatment of the human hepatocyte (HepG2) cell line caused a significant reduction in BODIPY staining, as a single agent or in combination with exogenously added lipid (Supplementary Fig. 7b).

Flux analysis of similarly treated cells showed that lipid challenge of hepatocytes increased their oxygen consumption rates (OCRs) and that this was accentuated by exposure to itaconate (Fig. 7d). The itaconate-mediated increase in lipid-induced OCR was completely abrogated in the presence of etomoxir, supporting the role for fatty acid oxidation as the source of lipid- and itaconate-induced increases in oxygen consumption in hepatocytes. In these experiments, itaconate treatment also caused an increase in glycolysis in hepatocytes, both as a single agent and in combination with lipid challenge (Fig. 7e). Both lipid and itaconate treatment significantly increased basal (Fig. 7f) and maximal OCR (Fig. 7g) although the effects in the combination treatment were not additive but rather primarily lipid driven. No difference in hepatocyte ATP-linked OCR was observed (Fig. 7h). Together, these data demonstrate the ability of exogenous itaconate to enhance oxidative phosphorylation of lipids and that this increase is not simply the result of glycolytic inhibition.

To further corroborate the link between fatty acid oxidation and itaconate exposure of hepatocytes, we treated cells with uniformly (U-^13^C16) labeled palmitate and confirmed that itaconate treatment results in increases in ^13^C-labeled TCA intermediates consistent with enhanced utilization of lipids. Treatment of hepatocytes with itaconate in the presence of isotopic labeled palmitate resulted in significant increases in isotopically labeled citrate (Fig. 7i), *cis*-aconitate (Fig. 7j) and TCA-cycle-derived metabolites such as aspartate (Fig. 7k), whereas the increases in labeled glutamate did not reach statistical significance (Fig. 7l). Although utilization of fatty acids was enhanced by itaconate, we detected no itaconate-dependent difference in fatty acid uptake in hepatocytes. Extracellular-labeled palmitate was equivalent between untreated and itaconate-treated hepatocytes (Supplementary Fig. 7c) and itaconate treatment had no effect on fatty acid uptake by hepatocytes as detected by fatty acid uptake assay (Supplementary Fig. 7d). Taken together, we conclude that itaconate enhances fatty acid utilization in hepatocytes through a mechanism independently of fatty acid uptake.

Although these data strongly imply a fundamental itaconate-induced alteration in metabolic processing, multiple reports have shown that itaconate is capable of eliciting substantial changes in transcription. Thus, to determine whether itaconate exerted transcriptional differences in hepatocytes that could account for the hyperlipidemic phenotype, we performed RNA-seq on lipid-challenged hepatocytes exposed or not to itaconate. We then interrogated expression of genes in the KEGG reference pathways for fatty acid metabolism^20^. Although the expression of 3 out of 35 genes (*Adh7*, *Echs1* and *Eci2*) in the fatty-acid reference pathway showed statistical differences between untreated and itaconate-treated hepatocytes and 4 genes (*Aldh1b1*, *Echs1*, *Eci2* and *Hadha*) were significantly changed between lipid and lipid + itaconate-treated hepatocytes, Gene Set Enrichment Analysis (GSEA) showed no significant difference in this pathway (Supplementary Fig. 8a). Moreover, despite previous reports implicating hepatic *Irg*1 induced by ischemia/reperfusion in the regulation of the Nrf2 antioxidant response^10^ and those suggesting itaconate as a key regulator of Nrf2 activity in macrophages^21^, we show that itaconate exposure of hepatocytes had only a minimal effect on Nrf2 target expression^22^. Out of 13 target genes, just one (Gsta3) showed significance upon itaconate treatment, but with <30% change between treatment groups, whereas 12 other genes showed no significant differences attributable to itaconate (Supplementary Fig. 8b).

**Fig. 8:**
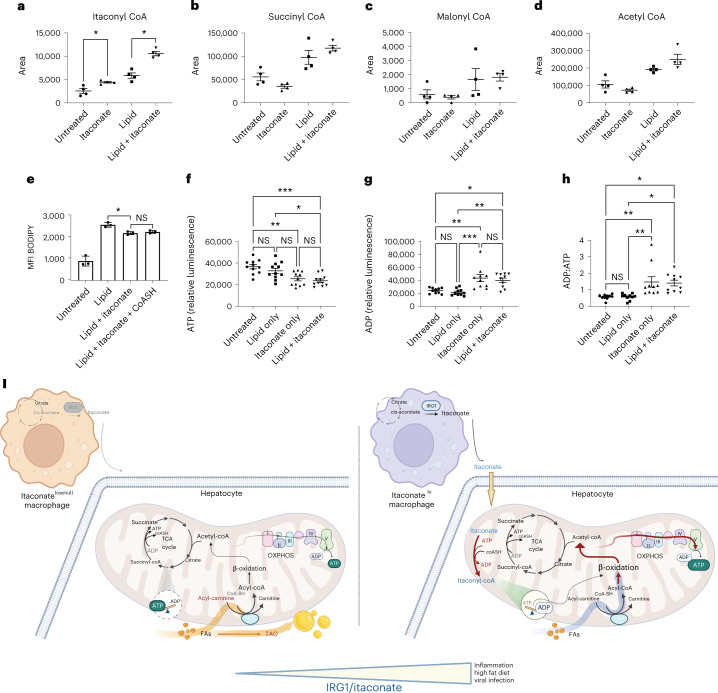
Itaconate suppression substrate-level phosphorylation could lead to compensatory β-oxidation. **a**–**d**, Hepatocytes were treated with 10 mM itaconate and/or 1:100 lipid. Levels of itaconyl-CoA (**a**) (*P = 0.049), succinyl-CoA (**b**), malonyl CoA (**c**) and acetyl-CoA (**d**) were measured by mass spectroscopy. The graphs depict triplicate wells from one experiment, representative of three experiments. **e**, BODIPY lipid staining in treated hepatocytes (*n* = 3). Some hepatocytes were treated in the presence of 0.5 mM CoASH (*P = 0.02; NS, not significant). **f**,**g**, Relative ATP (**f**) and ADP (**g**) levels were measured in treated hepatocytes (n = 11; *P = 0.011; **P = 0.003; ***P = 0.0003). **h**, ADP:ATP ratios in treated hepatocytes were calculated (*n* = 11; *P = 0.014; **P = 0.007). Data are presented as mean ± s.e.m. A two-sided ANOVA with multiple comparisons was used for statistical analyses. **i**, Schematic depicting net rebalancing of ADP:ATP levels in cells following metabolism of itaconate to itaconyl-CoA. Higher levels of itaconate result in a net loss of ATP due to itaconyl-CoA metabolism. Increases in ADP lead to a compensatory increase in β-oxidation, glycolysis and oxidative phosphorylation to restore cellular ATP levels that in turn reduces excess lipid levels. The image in **i** was created using BioRender.com under a full license to the National Cancer Institute (NCI). Source data

We next hypothesized that metabolic changes within itaconate-exposed hepatocytes might underlie enhancements in β-oxidation of lipid. As expected, itaconate treatment of hepatocytes resulted in increased levels of itaconyl-CoA (Fig. 8a). While lipid treatment increased all CoA species that we interrogated, we saw that itaconate had no effect on the levels of succinyl-CoA (Fig. 8b), malonyl CoA (Fig. 8c) or acetyl-CoA (Fig. 8d). Moreover, the addition of exogenous CoA (CoASH) had no effect on the BODIPY lipid staining in lipid + itaconate-treated hepatocytes (Fig. 8e). Taken together, these data argue against the CoA sink model in which itaconyl-CoA might regulate fatty acid metabolism and other CoA-dependent pathways by sequestering CoA^14^. We next interrogated whether itaconate could suppress mitochondrial substrate-level phosphorylation (mSLP) as proposed^23^. Indeed, itaconate-treated hepatocytes had significantly reduced ATP (Fig. 8f) and increased ADP (Fig. 8g) leading to significant shifts in their ADP:ATP ratios (Fig. 8h). This finding is consistent with the loss or consumption of ATP due to conversion of itaconate to itaconyl-CoA accompanied by increases in ADP. These data are also consistent with a model where reduced ATP levels due to a block in mSLP lead to compensatory increases in both β-oxidation and glycolysis, as we observed (Fig. 7), to facilitate the partial control of lipid levels and restore cellular ATP levels (schematized in Fig. 8i).

## Discussion

Altered metabolic pathways in the context of excess caloric intake can profoundly contribute to the development of fatty liver disease or hepatic steatosis, a chronic condition emerging as a major global health crisis^1,4^. The liver is the principal organ involved in the homeostasis of lipid metabolism, where tightly regulated biochemical pathways regulate the storage, biosynthesis, transport and elimination of fatty acids. Hepatic metabolism of fatty acids and triglycerides occurs primarily in hepatocytes^3^. Although the liver normally stores small amounts of triglycerides, in the context of excess caloric intake and disrupted lipid metabolism, triglycerides accumulate within hepatocytes, with severe impacts upon inflammation, cell death and fibrosis. Excess lipid accumulation can lead to altered lipid homeostasis that may manifest clinically as a spectrum of pathologies including obesity, type 2 diabetes and insulin resistance. Extensive steatosis may progress to liver failure, involving cirrhosis and hepatocellular carcinoma.

Mitochondrial β-oxidation represents a principal route for the processing of most fatty acids in hepatocytes^3^. Our study highlights an underappreciated role for metabolites such as itaconic acid to regulate fatty acid oxidation in hepatocytes. Itaconic acid, and *Irg1*, the gene responsible for itaconate synthesis, was discovered as a metabolite produced mainly by macrophages upon activation with a range of stimuli, including lipopolysaccharide, interferons and other Toll-like-receptor stimuli^5,8,12^. Itaconate is noteworthy because it has anti-inflammatory^21,24–26^ and potentially pro-tumorigenic^7,27^ properties and the understanding of its expression beyond myeloid immune cells has widened. In the liver, itaconate production by both hematopoietic cells and hepatocytes was responsible for protective responses following ischemia/reperfusion injury by stimulating the Nrf2-dependent protection of hepatocytes against oxidative stress^10^. Notably, we did not detect Nrf2 activation in hepatocytes treated with itaconate, a discrepancy possibly attributable to opposing effects on electrophilic stress and immune modulation between unmodified itaconate used in our studies and itaconyl derivatives such as 4-OI^19^. However, 4-OI administration was associated with a level of itaconate in the liver that potently reversed the hyperlipidemia and adiposity in high-fat-diet-fed *Irg1*-deficient mice. The finding that 4-OI administration did not have an effect in wild-type mice we believe highlights a poorly recognized homeostatic role for endogenous itaconate levels in the liver during lipid-related pathology.

Our data contribute to the expanding literature of myeloid-centric expression of *Irg1* and itaconic acid during pathogenic processes. In the mouse model of NAFLD that we employed, *Irg1* expression was seemingly confined to the myeloid compartment of cells and indistinguishable lipid phenotypes were observed in mice bearing both global and myeloid cell-specific deletion of *Irg1*. Although it remains possible that untreated hepatocytes have a low basal level of itaconate, inferred from the appearance of itaconyl-CoA in untreated and lipid-treated hepatocytes, we did not detect any increase in *Irg1* expression or itaconate production from lipid- or even LPS-treated hepatocytes and further found that *Irg1* deficiency specifically in hepatocytes has no effect on WD-induced dyslipidemia. Given the relative absence of *Irg1* expression and itaconate production by hepatocytes in the WD model we used, we believe that *Irg1* expression by hepatocytes following ischemia-reperfusion^10^ likely represents an important protective response that is specific to the injury achieved in that model. During the progression of fatty liver disease, resident KCs and local hematopoietic macrophages play critical roles in liver tissue remodeling and response to injury^28,29^. Hepatic macrophages can be activated by gut-derived endotoxins, fatty acids, cholesterol metabolites and molecules released from damaged hepatocytes whereby they can secrete proinflammatory cytokines, chemotactic factors and ROS; contribute to extracellular matrix degradation and tissue remodeling and promote angiogenesis. The frequency of activated macrophages is a predictive factor in the progression of human NAFLD^30^. Although activated macrophages also accumulate in adipose tissue and further fuel insulin resistance and hepatic lipid accumulation during NAFLD^31,32^, we found that Irg1 and itaconate expression was essentially confined to the macrophages of the liver, consistent with their unique roles in integrating signals from the gut/diet and as drivers of the progression of NAFLD to fibrosis and advanced steatohepatitis^31^. Although itaconate exerts protective effects against oxidative stress in hepatocytes, we believe that this is unlikely to be the case within the context of hepatic macrophages, as we and others showed that itaconic acid promotes lipid β-oxidation as a fuel source for oxidative phosphorylation that generates mitochondrial ROS contributable to oxidative stress in macrophage lineage cells^6,7^. Itaconate-deficient macrophages have defective oxidative phosphorylation and we cannot rule out contributions of hepatic macrophages to the lipid phenotype evident in *Irg1*^−/−^ mice. We believe it likely, however, that any such effects by macrophages are likely to be dwarfed by the direct influence of itaconate upon lipid metabolism by hepatocytes, due to their proficiency in lipid processing.

Of particular importance is the upregulated expression of *Irg1* and itaconate in human NASH. Moreover, we show that human hepatocytes exposed to itaconate show reductions in BODIPY lipid staining both in the presence and absence of exogenously added lipid. Taken together, we demonstrate the plausible role for itaconate in the regulation of lipid metabolism in livers of patients with NASH.

A role for Irg1 in metabolic disease was reported recently^33^. In that study, *Irg1* expression by adipocytes regulated inflammation and glucose homeostasis, although in that report hepatic expression of Irg1 was not investigated. As the central organ for the metabolism of fatty acids, our findings of *Irg1* expression in the liver in both a mouse model of NAFLD and human NASH cases, and the subsequent impacts upon hepatocyte metabolism, expand considerably our knowledge of the role by itaconate in lipid metabolism. Moreover, our data showing reduced lipid accumulation by the itaconate derivative, 4-OI, provides evidence of the therapeutic potential for targeting metabolites, including itaconate, for treating metabolic disease.

Given the excessive adiposity upon *Irg*1 deficiency, we propose a feedback system in which Irg1 expression by hepatic macrophages emerges as an important mechanism underlying the liver’s ability to metabolize lipid. Lipid homeostasis in KCs isolated from high-fat-diet-fed mice is already profoundly dysregulated, with prevailing bioactive and toxic lipid metabolites^34^. Our study supports the premise that *Irg1* expression is induced in NAFLD as an injury-response mechanism to metabolize excess lipid, although the potential for antimicrobial responses due to gut leakiness remains another possibility. In the absence of myeloid *Irg1* expression, liver lipid metabolism is impaired and potentially overwhelmed, resulting in exacerbated hyperlipidemia in association with the dramatic accumulation of carnitine species of fatty acids. The conjugation of carnitine to long-chain fatty acids is a prerequisite for their subsequent transfer into the mitochondria for β-oxidation^35^. As the liver becomes less able to process and oxidize lipid, the accumulating lipid becomes associated with increases in adiposity and a dramatic loss of macrophages in both adipose tissue and liver, presumably due to lipid-associated toxicity. During NAFLD, adipose tissue becomes dysfunctional, characterized by low-grade inflammation and the appearance of crown-like structures consisting of F4/80^+^ macrophages surrounding dying adipocytes^36,37^. In the liver, a similar structure has been described, consisting of F4/80^+^ macrophages forming a ring-like structure, generally surrounding dead or dying hepatocytes, a common feature of NASH^18^.

We hypothesize that the increased Irg1 expression and itaconate production during NASH serves as an important mechanism whereby excess lipid is metabolized by the liver; although *Irg1* expression is helpful, it is clearly not sufficient to prevent all aspects of the disorder. As modeled by *Irg1* deficiency under WD, exacerbated lipid accumulation results from the absence of this moderating metabolic pathway; however, there exists an apparent dichotomy in *Irg1* expression in the liver during the WD model. Azzimato et al. reported recently that *Irg1* expression was reduced in 9-week WD-fed mice and suggested that this loss facilitated disease^38^. Our data do not reveal a substantial drop in *Irg1* early in disease, but rather show increased expression of the enzyme later in WD feeding. These findings can be reconciled by a model where constitutively *Irg1*^*+*^ KCs are lost early in WD feeding followed by subsequent upregulation of Irg1 expression in F4/80^+^ macrophages as the resident macrophages undergoing cell death are replaced by newly recruited inflammatory macrophages^28,39^. Indeed, the single-cell transcriptomic analysis of NAFLD developed by Guilliams et al. similarly revealed *Irg1* expression was notably upregulated in response to 12–36 weeks of WD feeding in the CLEC4f^−^ macrophages recruited from the circulation but not in resident KCs^17^ (Fig. 2a). In that study, WD feeding elicited the loss of KCs due to apoptosis concomitant with the dramatic appearance of *Irg1*^+^ macrophages from the circulation. Although recruited macrophages undergo reprogramming in the liver environment and eventually take up characteristics of resident KCs^39^, *Irg1*^+^ KCs remain undetectable even as late as 36 weeks of WD feeding, indicating that hepatic *Irg1* expression in our study is predominantly from macrophages of circulatory origin. Neutrophils also accumulate during NASH and contribute to hepatocyte injury^40^ and we cannot rule out contributions of *Irg1*^+^ neutrophils toward lipid metabolism in the liver; however, *Irg1* expression by neutrophils is independent of WD feeding, neutrophil cell numbers across all cellular zones of the liver are dwarfed by those of monocytes and macrophages in the steatotic liver^17^ and we show that itaconate expression is confined to F4/80-sorted macrophages. Irg1 was coexpressed with IFNAR^17,28^. We found that *Irg1* upregulation in the liver is dependent on IFNAR expression but independent of Stat1 expression suggesting that non-canonical type I interferon signaling or potentially Stat2 homodimers may be important to the upregulated *Irg1* expression in the liver following WD^18^.

We show that itaconate, at physiologically relevant concentrations, significantly reduces lipid accumulation in primary hepatocytes and that this is accompanied by an increase in oxidative phosphorylation. The itaconate-mediated increases in OXPHOS were abrogated in the presence of etomoxir, supporting the premise that β-oxidation of lipids drives the increase in hepatocyte OXPHOS. Itaconate has been shown to inhibit aerobic glycolysis^12,41–43^; however, this was described in actively dividing cells and not for relatively quiescent cells such as cultured hepatocytes^44^, where we also did not observe accumulations or alterations in levels of succinate or other glycolytic intermediates due to itaconate treatment. Although succinate was shown to accumulate in itaconate-exposed Huh7 cells^14^, we detected no change in succinate levels in primary hepatocytes that could account for the ability of itaconate to reduce lipid accumulation. Shifts away from glycolysis and toward OXPHOS could possibly explain the increases in OXPHOS that we observed; however, we found no such evidence; in fact, itaconate treatment caused an increase in the overall metabolic activity of hepatocytes by increasing OXPHOS as well as glycolysis in a lipid-dependent manner. Although multiple reports have shown itaconate to be capable of eliciting substantial changes in transcription^21,24^, despite substantial metabolic alterations during itaconate exposure, we found no evidence of significant transcriptional alterations in fatty-acid oxidation or oxidative phosphorylation KEGG pathways in hepatocytes. Thus, we believe regulation of lipid oxidation by itaconate is due to metabolic, rather than transcriptional, effects.

We demonstrate the ability of itaconate to promote fatty-acid oxidation in hepatocytes. Mechanistically our data support the purported role of itaconate in suppressing mitochondrial substrate-level phosphorylation (mSLP)^23^. We and others, find that cellular itaconate is effectively activated to itaconyl-CoA, presumably via succinyl-CoA ligase. Within the TCA, this enzyme produces ATP while converting succinyl-CoA to succinate and free CoA. Itaconate subjugates this enzyme for the reverse reaction and in the process consumes ATP and CoA, yielding ADP and itaconyl-CoA. Our supplementation of CoA showed little effect on lipids, ruling out itaconate as a CoA sink^14^. Rather, we find that addition of itaconate increases ADP in hepatocytes in conjunction with the appearance of itaconyl-CoA. Thus, we propose a model where a reduction of ATP levels, driven by a loss of mSLP and subsequent consumption of ATP during activation of itaconate, leads to a compensatory increase in β-oxidation and glycolysis. This fatty-acid-dependent β-oxidation, our data suggest, facilitates the partial control of lipid levels in the hepatocytes of mice fed a high-fat diet and blunts metabolic disorder.

Although our study identifies an important mechanism whereby itaconate promotes lipid oxidation by hepatocytes, it is important to consider the potential for itaconate-mediated regulation of other resident liver cells. For example, hepatic stellate cells (HSCs) play important roles in liver fibrosis and store, and are potential sources of, bioactive lipids^45^. Although we have not directly assessed the role of HSCs here, it is possible that itaconate regulates lipid oxidation in HSCs in addition to hepatocytes and that this subset substantially contributes to increased consumption of lipid induced by itaconate. Additional HSC-targeted studies would be needed to fully determine whether itaconate regulates HSC function/fibrosis and whether HSC involvement contributes to itaconate-driven lipid metabolism in the liver.

In summary, we identify an important connection between immunometabolism and lipid metabolism in the liver. Itaconate production in human NASH and mouse models of fatty liver disease highlights the necessity of further investigation of mechanisms whereby this key immunometabolite regulates lipid oxidation by hepatocytes and potentially other cells. These findings suggest that interventions to alter itaconate levels may hold therapeutic potential to regulate metabolic disease and dyslipidemia.

## Methods

### Mice

Wild-type C57BL/6J mice were obtained from The Jackson Laboratory and bred at the NCI Frederick Cancer Research and Development Center. *Irg1*^−/−^ mice were obtained from M. Diamond (Washington University School of Medicine) and backcrossed ten generations onto C57BL/6J background. *LysM*^Cre^ or *Alb*^Cre^ mice on a C57BL/6J background at NCI Frederick breeding core were crossed with *Irg1*^fl/fl^ mice (generously provided by M. Diamond) to generate *LysM*^Cre^ × *Irg1*^fl/fl^ and *Alb*^Cre^ × *Irg1*^fl/fl^ mice, respectively. The C57BL/6J background of all mice was confirmed by DartMouse (DartMouse Laboratory; under National Institutes of Health (NIH) grants 1S10OD021667-01 and 5P30CA023108). Male mice were used at 8–12 weeks of age in accordance with an approved NCI Frederick Animal Care and Use Committee protocol. Male mice were used because female rodents are relatively resistant to hyperphagia and weight gain in response to a high-fat diet, and to normalize for sex-specific differences in insulin responses for glucose homeostasis. Mice were housed on a 12-h dark–light cycle at 68–79 °F and 30–70% humidity. The mouse model of NAFLD was induced by feeding mice ad libitum with RD western diet (Research Diets) supplemented with 10% sucrose in drinking water for 12 weeks as described^15,16,46,47^. Some mice were treated three times per week intraperitoneally (i.p.) with 50 mg kg^−1^ 4-OI (Cayman Chemical) in 40% cyclodextrin/PBS (or 40% cyclodextrin/PBS as vehicle control) as described^19,41^ for 12 weeks.

### Human tissue and cells

Frozen liver tissue from 16 de-identified patients with NASH and 10 non-NASH controls were provided through the Clinical Biospecimen Repository and Processing Core of the Pittsburgh Liver Research Center. The non-NASH controls had diagnoses of hemangioma, focal nodular hyperplasia, hepatic adenoma, hepatocellular carcinoma and benign liver mass. Samples were homogenized in lysis buffer or 80% methanol and processed as described below for qPCR and metabolomics, respectively. The hepatocyte cell line of human origin (HepG2) was from the American Type Culture Collection (cat. no. HB-8065).

### Histological analysis/quantitation

Liver tissue sections were fixed in 4% buffered formalin and embedded in paraffin or snap frozen in OCT compound. Fixed, paraffin-embedded tissue sections were incubated with antibody to F4/80 (1:100 dilution; clone BM8, eBioscience), followed by horseradish peroxidase- or alkaline phosphatase-conjugated secondary antibodies (1:100 dilution; rabbit anti rat IgG; Vector Labs). Frozen tissues were stained in ORO (Sigma-Aldrich). At least five non-overlapping fields per sample were imaged using Aperio eSlide Manager and ImageScope software (Leica Biosystems) and quantitation was performed using ImageJ software using thresholding of object sizes to eliminate debris. Average ORO droplet size was calculated by dividing the sum of stained area by the total number of droplets in the same field.

### Isolation of F4/80^+^ macrophages

Liver tissues were homogenized in 200 U mg^−1^ type 1 collagenase (Worthington Biochemical), 1 mg ml^−1^ dispase 2 (Sigma) and 0.5 mg ml^−1^ DNase 1 (Sigma) using a GentleMacs Tissue Dissociator (Miltenyi Biotec). The single-cell suspension was filtered through a 70-μM filter. The washed cells were incubated with biotinylated anti-F4/80 antibody (1:25 dilution; clone BM8; BioLegend) followed by magnetically coupled streptavidin microbeads (Miltenyi Biotec). F4/80^+^ cells were positively selected by magnetic separation (Miltenyi Biotec) to >97% purity. The flow-through fraction containing the non-macrophages was saved for analysis.

### Quantitative PCR

Total RNA was isolated using High Pure RNA Isolation kits (Roche Diagnostics). RNA was reverse transcribed using the High Capacity cDNA Archive kit (Applied Biosystems). Murine and human *Irg1* (Mm01224532_m1 and Hs00985781_m1, respectively) and *IL6* (Hs00985639_m1) expression was examined using validated gene expression assays (Applied Biosystems). Briefly, 10 ng cDNA was put in a final volume of 20 µl containing 10 µl Taqman Universal PCR mix and 1 µl primer/probe gene expression assay. All samples were run on an ABI 7300 real-time PCR system and analyzed using the ΔΔCT method^48^. Gene expression was normalized to the level of the housekeeping gene *HPRT* (Mm01545399_m1) or *GAPDH* (Hs02758991_g1).

### Metabolomics

For detection of itaconate and TCA intermediates, cell pellets (~1 × 10^6^ cells) were washed, resuspended in 80% methanol and stored overnight at −80 °C. Liver tissues were weighed and homogenized in a GentleMacs Dissociator (Miltenyi Biotec) with 80% methanol/0.1 M formic acid. The molar concentration of itaconate in the liver was approximated as described^49^. Serum was spun using serum separator tubes. For medium and serum samples, 50 μl was mixed with 100% methanol/0.1 M formic acid. For each, phase separation was achieved by centrifugation at 4 °C. The methanol:water phase containing polar metabolites was dried using a vacuum concentrator. Targeted measurements on the resuspended metabolite samples were performed through electrospray ionization mass spectrometry (ESI-LC–MS/MS) analysis as described^50^. A standard curve of itaconic acid (Millipore Sigma) was used for quantitation.

For broad metabolomics analysis, cells were washed with cold 150 mM ammonium acetate and resuspended in cold 80% methanol/0.1 M formic acid. Phase separation and lyophilization of samples was performed as described above. Lyophilized samples were reconstituted in 50% acetonitrile and analyzed in an Agilent 1290 Infinity II ultra-high performance liquid chromatography (UHPLC) system coupled with an Agilent 6546 quadrupole time-of-flight (Q-TOF) mass spectrometer. Acquired data were analyzed using Agilent MassHunter Profinder 8.0, which performed data processing of >500 target metabolites derived from an in-house accurate mass retention time metabolite standard library^51^.

For ^13^C-tracing studies, cells were incubated in DMEM supplemented with 10% dialyzed FBS, 2 mM l-glutamine and BSA-conjugated palmitate U-^13^C16 (Cambridge Isotope Laboratories) at a final concentration of 200 μM for 4 h^52^. Cells were processed as described above. Isotopolog analysis of acquired negative MS data was performed with MassHunter Profinder v.8.0, which performed simultaneous data processing and quantitation of ^13^C incorporation in a list of target metabolites derived from an in-house accurate mass retention time metabolite standard library. Extracted isotopolog abundancies were corrected for their natural isotope abundance. M + 0 to M + *n* indicate the different mass isotopologs for a given metabolite with *n* carbons, where mass increases due to ^13^C-labeling.

### Lipidomics

Cell pellets (~1 × 10^6^ cells) were resuspended in methanol, extracted with chloroform and centrifuged for 5 min at 4 °C. Dried lipid extracts were reconstituted with 50 μl methanol/chloroform (9:1 v/v). Analysis was carried out on an Agilent 1290 Infinity II UHPLC system coupled with an Agilent 6546 quadrupole time-of-flight (Q-TOF) mass spectrometer. Final curation of annotated lipids was performed with Agilent MassHunter Profinder (v.10.0.2) as described^53^. Agilent MassHunter Mass Profiler (Professional v.15.1) was used for statistical analysis.

### Acyl-CoA analysis

Acyl-CoAs were extracted by resuspension of the cell pellets in 10% trichloroacetic acid, sonication, centrifugation at 17,000*g* 10 min 4 °C and extraction of the supernatant by 80% methanol (final concentration) containing 25 mM ammonium acetate. Supernatant from further centrifugation at 17,000*g* 10 min 4 °C was dried using a vacuum concentrator and then resuspended in 50 µl water. Liquid chromatography–high-resolution mass spectrometry analysis was conducted as previously described^54^ on a Q Exactive Plus operating in positive-ion mode coupled to a Vanquish Duo LC. Analysts were blinded to sample identity.

### Biochemical analyses on serum and tissue samples

Blood was collected at the indicated times and placed into serum separator tubes that were spun 12,000*g* for 5 min and stored frozen until use. Serum insulin levels were determined using a commercial ELISA (Cayman Chemical). Free fatty acids were determined using an enzyme-based assay kit (Abcam). A triglyceride colorimetric assay kit (Cayman Chemical) was used to determine triglycerides in serum samples or homogenized tissues. The EnzyLight ADP/ATP ratio assay kit and QuantiChrom fatty acid uptake assay kit were from BioAssay Systems.

For glucose tolerance testing, mice were fasted for 16 h. Blood glucose levels were tested using a commercially available blood glucose meter and test strips (CVS Health). Baseline glucose levels were read and then mice were challenged i.p. with 2 g kg^−1^ sterile glucose in 100 μl saline. For insulin tolerance testing, 5 h fasted mice were injected i.p. with a single dose of insulin (0.5 U kg^−1^). Blood glucose was read every 15 min for 2 h.

### Preparation of hepatocytes

Hepatocytes were isolated from freshly isolated mouse livers (wild-type mice) as described^44^. Unless indicated otherwise, cultured hepatocytes were treated overnight in complete medium (DMEM supplemented with 5% FBS; 1× insulin-transferrin-selenium, Thermo Fisher Scientific) with sodium itaconate (10 mM unless otherwise noted) in the presence or absence of a dilution (1:10 unless otherwise noted) of sterile, chemically defined lipid mixture 1 (2 μg ml^−1^ arachidonic acid; 10 μg ml^−1^ each of linoleic, myristic, oleic, palmitic and stearic acids; and 0.22 mg ml^−1^ cholesterol; Sigma-Aldrich).

### Extracellular flux analysis

Isolated hepatocytes were seeded at 1.7 × 10^4^ cells per 96-well in complete medium and treated as described overnight. Plated cells were washed gently with PBS and incubated with Seahorse assay medium supplemented with 2 mM glutamine and 25 mM glucose for 1 h at 37 °C with no CO_2_. Extracellular flux analysis was performed at 37 °C with no CO_2_ using the XF-96 analyzer (Seahorse Bioscience) per the manufacturer’s instructions. Port additions and times were used as indicated in the figures.

### Flow cytometric analysis

Cells (1 × 10^6^) were incubated in cell staining buffer (0.1% BSA and 0.1% sodium azide) containing 250 µg ml^−1^ 2.4G2 ascites for 15 min. Cells were stained with either fluorescently conjugated antibodies to F4/80 (1:25 dilution; clone BM8) or Ly6 (1:100 dilution; clone 1A8) or the fluorescent probe BODIPY 493/503 (Thermo Fisher Scientific) for 20 min. After washing, the labeled cells were analyzed on an LSR II flow cytometer using FACS-DIVA software (Becton Dickinson) and FlowJo software (v.10).

### Bulk RNA sequencing

Total RNA was isolated from treated hepatocytes using a PureLink RNA Mini kit with on-column DNase digestion (Invitrogen). All samples met quality control of RNA integrity number > 8.0 using a Bioanalyzer. Then, 500 ng total RNA was used via the stranded mRNA ligation kit (Illumina) as the input to an mRNA capture, cDNA synthesis and standard Illumina library prep before sequencing on the NextSeq 2000 instrument (Illumina). HiSeq Real Time Analysis software (RTA v.3.9.25) was used for processing raw data files. Illumina Bcl2fastq v.2.20 was used to demultiplex and convert binary base calls and qualities to fastq format. Trimmed sequencing reads (Cutadapt v.1.18) were mapped to mouse reference genome mm10 and annotated using GENCODE STAR aligner (v.2.7.0f) with two-pass alignment. Mapping statistics were calculated using Picard v.2.18.26. RSEM (v.1.3.1) was used for gene and transcript quantification based on GENCODE annotation files.

For RNA-seq data processing, raw counts were uploaded to the Partek Flow platform and all analysis was carried out using default parameters. Counts were normalized and filtered to remove low expressed features (<1.0). Batch removal was carried out using a general linear model. Data were visualized using principal-component analysis and differential gene expression analysis was carried out using ANOVA testing comparing different treatment conditions. GSEA was performed as described^55^. Briefly, raw counts were normalized with the median ratio method from DESeq2. Batch effects were removed and visualized using principal-component analysis. Normalized and batch-corrected counts were imported to the GSEA software v.4.2.3 (Broad Institute). Gene sets were obtained from MSigDB (Broad Institute) or generated de novo in a gene matrix transposed file format. Analysis was run using default parameters, with the exception of ‘Min size,’ which was reduced to 2.

### Statistical analysis

Statistical differences between groups and any areas under the curve were analyzed using GraphPad Prism software. Two-way ANOVA with multiple comparisons was used unless otherwise noted in the corresponding figure legend. For non-normally distributed data between two groups (human NASH study), a Mann–Whitney *U*-test was used. Multiple *t*-tests (one per row) were used for large datasets of lipidomics or metabolomics comparing two treatment groups. Significance is indicated by *P < 0.05; **P < 0.01; ***P < 0.001; and ****P < 0.0001 values.

### Reporting summary

Further information on research design is available in the Nature Portfolio Reporting Summary linked to this article.

## Supplementary information

Supplementary Information Supplementary Figs. 1–8 with figure legends.

Reporting summary

Supplementary Data Source Data for supplementary figures.

## Data Availability

Source data are provided with this paper. The original datasets used in the RNA-seq analysis can be accessed at the National Center for Biotechnology Information archived under Gene Expression Omnibus accession code GSE227900. Mass spectrometry metabolomics and lipidomics data for Figs. 1 and 7 and Supplementary Figs. 5 and 7 are provided in the Supplementary Information and accessible via FigShare (10.6084/m9.figshare.21183331). Source data are provided with this paper.

## Source data

Source Data Fig. 1 Statistical source data.
Source Data Fig. 2 Statistical source data.
Source Data Fig. 3 Statistical source data.
Source Data Fig. 4 Statistical source data.
Source Data Fig. 5 Statistical source data.
Source Data Fig. 6 Statistical source data.
Source Data Fig. 7 Statistical source data.
Source Data Fig. 8 Statistical source data.
