# Supplementary material for: Itaconic acid underpins hepatocyte lipid metabolism in non-alcoholic fatty liver disease in male mice
Source: Nat Metab. 2023 Jun 12;5(6):981–95. doi: 10.1038/s42255-023-00801-2 (PMC10290955; doi:10.1038/s42255-023-00801-2)
Supplement: Supplementary file 1 — Supplementary Figs. 1–8 with figure legends. [file 42255_2023_801_MOESM1_ESM.pdf]

nature metabolism

Article

https://doi.org/10.1038/s42255-023-00801-2

Itaconic acid underpins hepatocyte lipid metabolism in non-alcoholic fatty liver disease in male mice

In the format provided by the authors and unedited

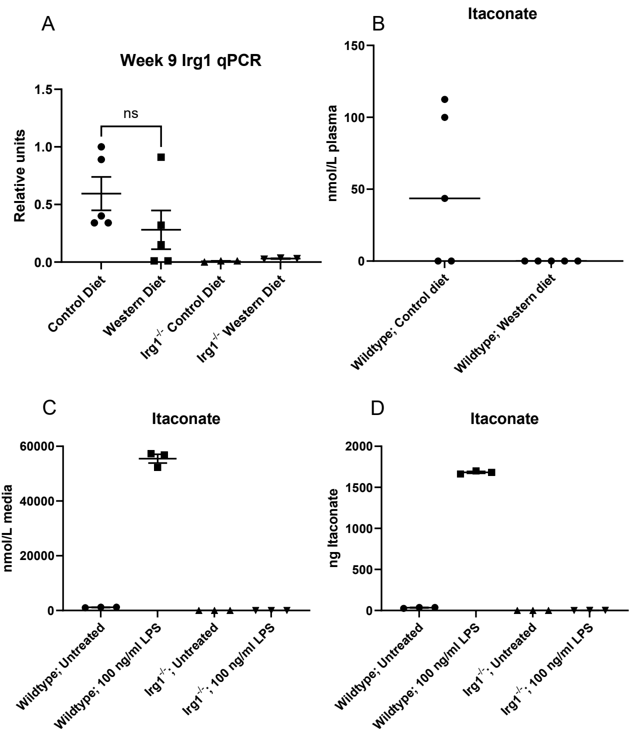

Supplemental Figure 1. Irg1 expression and itaconate production in week 9 WD and LPS treated hepatocytes. (A) Whole liver lobes were harvested on week 9 following control or WD feeding of the indicated mice. Murine Irg1 gene expression was quantified in each sample using qPCR (n=5 mice/group; ns= not significant by two-sided ANOVA). (B) Systemic levels of itaconate in the blood of control diet and WD fed mice (n=5; differences were not significant). (C) Extracellular and (D) intracellular itaconate production was quantified in $2x10^6$ plated wildtype BMM treated overnight with 100 ng/ml LPS (n=3). No itaconate was produced using BMM from *Irg1*$^{-/-}$ mice.

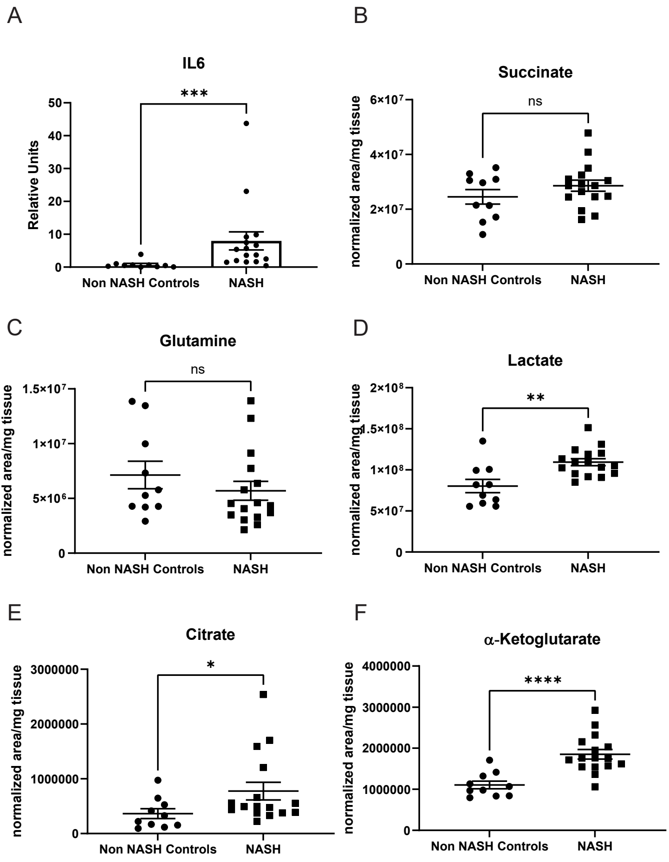

Supplemental Figure 2. Cytokine/metabolite expression in human NASH. (A) Human IL-6 gene expression was quantified in patient liver samples using qPCR (n=10 non-NASH controls and 16 NASH cases; *** p=0.0001). Results for *Irg1* are shown as the relative fold change over non-NASH controls. (B) Succinate, (C) Glutamine, (D) Lactate, (E) Citrate and (F) α-Ketoglutarate expression in human non-alcoholic steatohepatitis (NASH) livers by mass spectrometry (* p=0.027; ** p=0.003; **** p<0.0001). Results for human liver samples from 10 non-NASH controls and 16 NASH cases were normalized for mg liver tissue. A two-sided Mann-Whitney statistical comparison was performed between each group.

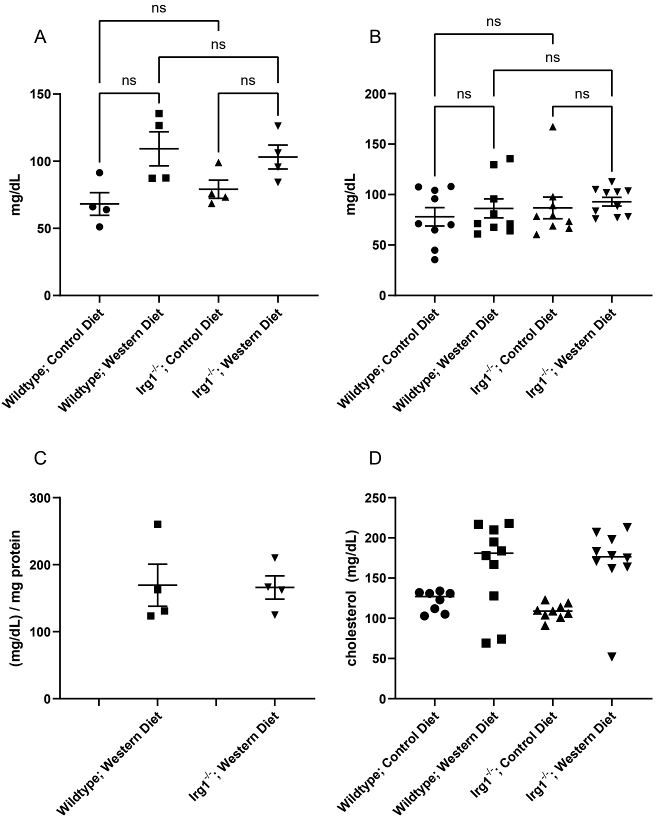

Supplemental Figure 3. **Serum and adipose triglycerides are unaltered in *Irg1$^{-/-}$* mice on Western diet.** Serum triglycerides were evaluated at (A) 6 weeks (n=4/group) and (B) 12 weeks of control or Western diet (n=8/group). (C) Triglycerides in adipose tissue were evaluated in Wildtype and Irg1-/- mice after 6 weeks of Western diet only (n=4 mice/group. (D) Serum cholesterol was evaluated from the indicated mice after 12 weeks of feeding (n=8 mice/group). For all panels, no significance was observed between wildtype and *Irg1$^{-/-}$* mice (two-sided ANOVA).

A

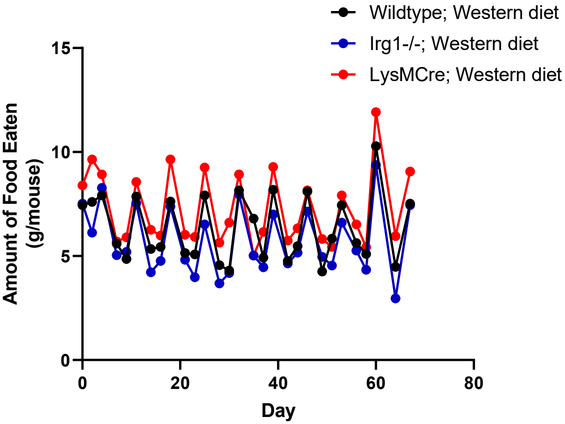

Supplemental Figure 4. Equivalent food consumption between wildtype, Irg1$^{-/-}$ and Irg1$^{fl/fl}$ x LysM^{Cre} mice. The amount of food/cage normalized for the number of mice/cage was weighed 3x/weekly. All three mouse strains consumed an equivalent amount of food over 12 weeks of WD feeding. The valleys in the graph are due to weekends without food replenishment. The graph depicts results from one experiment (n=5 mice/group) and is representative of 3 experiments.

A

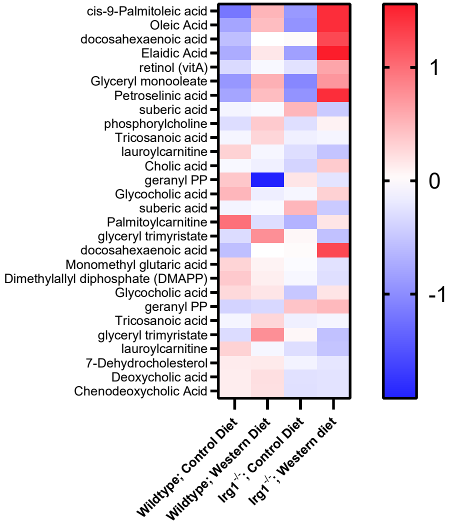

B

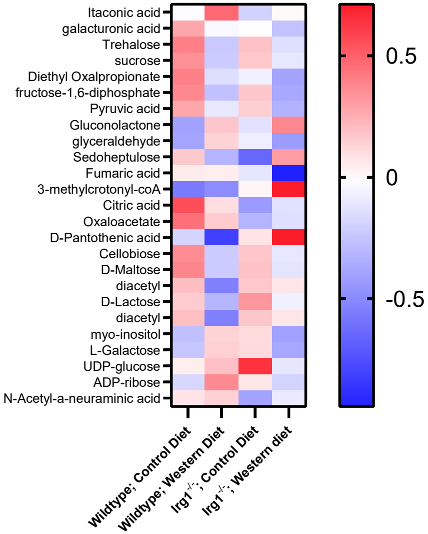

Supplemental Figure 5. Broad metabolomics analysis of F4/80$^+$ macrophages from wildtype and Irg1$^{-/-}$ mice following control or Western diet feeding. F4/80$^+$ macrophages were isolated from the livers of control or Western diet fed mice. Broad metabolomics was used to analyze (A) lipid metabolites and (B) energetic metabolites. For each metabolite, a ratio was computed by dividing the metabolite level by the relative mean for all 10 samples (5 wildtype and 5 Irg1$^{-/-}$). The heat map depicts $\log_2$ transformed ratios for metabolites.

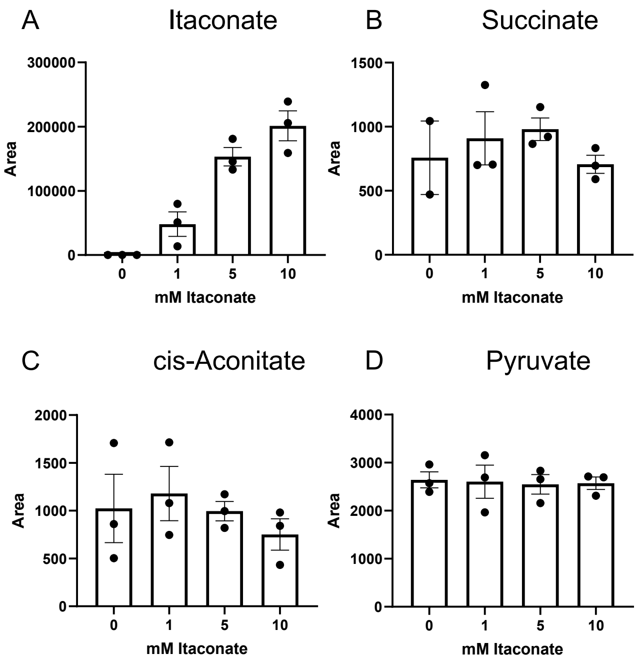

Supplemental Figure 6. Dose dependent uptake of itaconate by hepatocytes. Hepatocytes were treated with the indicated dose of itaconate overnight. Hepatocytes were analyzed for itaconate and TCA intermediates by mass spectrometry. (A) Dose-dependent uptake of itaconate by hepatocytes. Itaconate treatment had no effect on the accumulation of (B) succinate, (C) cis-aconitate or (D) pyruvate. The results from three hepatocyte cultures are shown; similar findings were observed in 3 separate experiments.

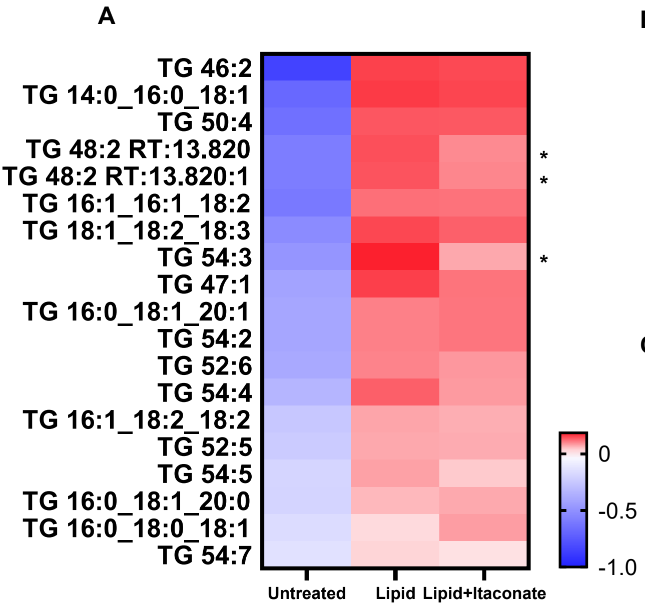

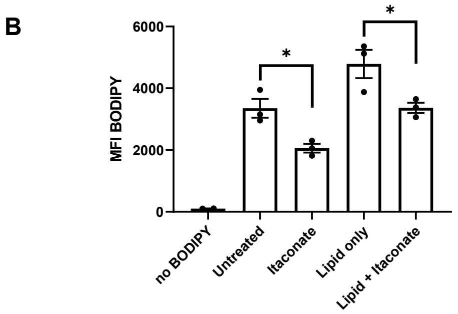

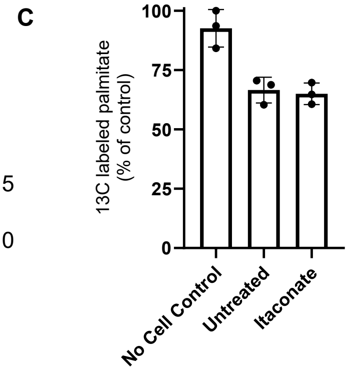

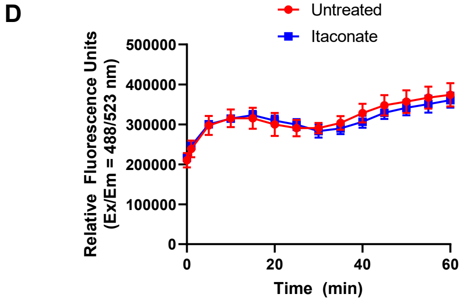

Supplemental Figure 7. Lipidomics of itaconate-treated hepatocytes in the presence of lipid. (A) Hepatocytes were treated overnight with 10 mM itaconate in the presence of 1:10 dilution of lipid mixture. Targeted lipidomics showing triglycerides between lipid and lipid+itaconate treated hepatocytes. For each metabolite, a ratio was computed by dividing the metabolite level by the relative mean for all 9 samples (3 samples per treatment group). The heat map depicts $\log_2$ transformed ratios for metabolites (*p=0.04). (B) HepG2 cells were treated overnight with itaconate alone or in the presence of lipid. The graph shows BODIPY staining from one experiment of triplicate wells/treatment that is representative of 2 experiments (* p=0.026). (C) Extracellular 13C16 labeled palmitate in hepatocytes (n=3). Hepatocytes were cultured overnight in 1:10 lipid mix and the presence or absence of 10 mM itaconate. U-13C16 labeled palmitate was added for the final 4 hr of culture. Supernatants were extracted as described and the extracellular levels of labeled palmitate was measured by mass spectrometry. The level of palmitate in the media without any cells was set to 100% and the percentage of labeled palmitate in the cultures of treated hepatocytes shown as relative to this control. (D) Fatty acid uptake was measured in untreated and 10 mM itaconate treated hepatocytes as described in Methods (n=4/group).

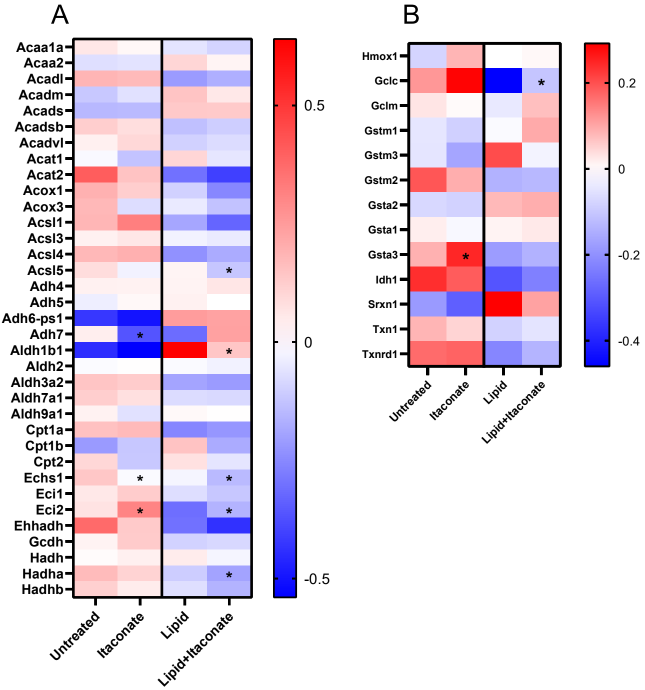

Supplemental Figure 8. Transcriptomics of itaconate-treated hepatocytes. Hepatocytes were treated overnight with 10 mM itaconate and/or 1:10 dilution of lipid mixture. Total RNA was isolated (n=4/group) and global gene expression was analyzed by bulk RNA seq. For each gene, a ratio was computed by dividing the gene expression level by the relative mean for all 16 samples (4 per treatment group). Heatmaps depict the log2 transformed ratios for (A) KEGG reference pathway genes for fatty acid metabolism and (B) Nrf2 gene targets. Two-way multiple t tests (one per row) were used to determine significance between two groups (untreated versus itaconate treated cells) and (lipid versus lipid+itaconate treated cells). Asterisks denote those genes significantly different between the corresponding treatment groups (* $p<0.05$).
