# Supplementary material for: Itaconic acid underpins hepatocyte lipid metabolism in non-alcoholic fatty liver disease in male mice
Source: Nat Metab. 2023 Jun 12;5(6):981–95. doi: 10.1038/s42255-023-00801-2 (PMC10290955; doi:10.1038/s42255-023-00801-2)
Supplement: Supplementary file 2 — Reporting summary [file 42255_2023_801_MOESM2_ESM.pdf]

nature portfolio

Corresponding author(s): McVicar, DW and Weiss, JM

Last updated by author(s): Mar 24, 2023

Reporting Summary

Nature Portfolio wishes to improve the reproducibility of the work that we publish. This form provides structure for consistency and transparency in reporting. For further information on Nature Portfolio policies, see our Editorial Policies and the Editorial Policy Checklist.

Statistics

For all statistical analyses, confirm that the following items are present in the figure legend, table legend, main text, or Methods section.

n/a	Confirmed	
☐	☒	The exact sample size (*n*) for each experimental group/condition, given as a discrete number and unit of measurement
☐	☒	A statement on whether measurements were taken from distinct samples or whether the same sample was measured repeatedly
☐	☒	The statistical test(s) used AND whether they are one- or two-sided<br>*Only common tests should be described solely by name; describe more complex techniques in the Methods section.*
☐	☒	A description of all covariates tested
☒	☐	A description of any assumptions or corrections, such as tests of normality and adjustment for multiple comparisons
☐	☒	A full description of the statistical parameters including central tendency (e.g. means) or other basic estimates (e.g. regression coefficient) AND variation (e.g. standard deviation) or associated estimates of uncertainty (e.g. confidence intervals)
☐	☒	For null hypothesis testing, the test statistic (e.g. *F*, *t*, *r*) with confidence intervals, effect sizes, degrees of freedom and *P* value noted<br>*Give P values as exact values whenever suitable.*
☒	☐	For Bayesian analysis, information on the choice of priors and Markov chain Monte Carlo settings
☒	☐	For hierarchical and complex designs, identification of the appropriate level for tests and full reporting of outcomes
☒	☐	Estimates of effect sizes (e.g. Cohen's *d*, Pearson's *r*), indicating how they were calculated

Our web collection on statistics for biologists contains articles on many of the points above.

Software and code

Policy information about availability of computer code

Data collection	RTA version 3.9.25, Bcl2fastq version 2.20, Cutadapt version 1.18, STAR version 2.7.0f, Picard version 2.18.26, RSEM version 1.3.1, FACS-DIVA (Becton Dickinson), Aperio eSlideManager (Leica)
Data analysis	GraphPad Prism 9, GSEA version 4.2.3, R version 4.2.0, RStudio version 2022.07.1 build 554, Partek Flow build version 10.0.22.0828, FlowJo version 10, ImageScope version 12.4.6.7001, ImageJ version 1.53e

For manuscripts utilizing custom algorithms or software that are central to the research but not yet described in published literature, software must be made available to editors and reviewers. We strongly encourage code deposition in a community repository (e.g. GitHub). See the Nature Portfolio guidelines for submitting code & software for further information.

Data

Policy information about availability of data

All manuscripts must include a data availability statement. This statement should provide the following information, where applicable:

- Accession codes, unique identifiers, or web links for publicly available datasets
- A description of any restrictions on data availability
- For clinical datasets or third party data, please ensure that the statement adheres to our policy

The metabolomics and lipidomics data generated in this study are accessible via Figshare (https://doi.org/10.6084/m9.figshare.21183331). The RNA sequencing dataset generated during and/or analyzed during the current study are deposited and can be accessed under GEO accession code GSE227900.

Human research participants

Policy information about studies involving human research participants and Sex and Gender in Research.

Reporting on sex and gender	Sex was not considered in the study design. Frozen liver tissues from the Pittsburgh Liver Research Center contained samples from both male and female patients.
Population characteristics	Frozen liver tissue from deidentified 16 patients with non-alcoholic steatohepatitis (NASH) and 10 non-NASH controls were provided through the Clinical Biospecimen Repository and Processing Core of the Pittsburgh Liver Research Center. The non-NASH controls had diagnoses of hemangioma, focal nodular hyperplasia, hepatic adenoma, hepatocellular carcinoma and benign liver mass. No covariant relevant characteristics (age, past diagnosis, treatment categories, genotype) were known as all samples were coded with an anonymized participant number.
Recruitment	All human tissues were provided through the Clinical Biospecimen Repository and Processing Core of the Pittsburgh Liver Research Center. All frozen tissue was from deidentified patients.
Ethics oversight	The NIH/NCI Intramural Board has determined that our work falls under non-human subjects research (NHSR) and exempted from IRB review.

Note that full information on the approval of the study protocol must also be provided in the manuscript.

Field-specific reporting

Please select the one below that is the best fit for your research. If you are not sure, read the appropriate sections before making your selection.

☒ Life sciences ☐ Behavioural & social sciences ☐ Ecological, evolutionary & environmental sciences

For a reference copy of the document with all sections, see nature.com/documents/nr-reporting-summary-flat.pdf

Life sciences study design

All studies must disclose on these points even when the disclosure is negative.

Sample size	No statistical methods were used to predetermine sample size. Sample sizes were determined based on prior studies with similar experimental design and on the known variability of the assay, balancing statistic robustness with resource availability. The number of mice that were used for each experiment was determined to give reliable and robust conclusions. The number of mice per group for each experiment are indicated in figure legends, and depicted as individual dots in graphs. Animal experiments were repeated at least three times, and the results from all mice pooled. For quantitation of digital images, at least 5 non-overlapping fields/animal were captured, for a total of at least 25 images per treatment per experiment which ensured broad coverage of histologic samples.
Data exclusions	No data were excluded from the dataset
Replication	All experiments were reproducible. The biological replicates or independent experiments are presented in each figure legend. The mice used for experiments were from multiple litters.
Randomization	Mice were randomly allocated into experimental and control groups by animal technical staff. Randomization was not possible for in vitro experiments or the human liver cases, which were grouped according to clinical diagnosis.
Blinding	Investigators were blinded as to the experimental groups mice were allocated. Only animal technical staff who were performing the injections were aware of the allocation of mice to each group. Mouse tissue weights and blood/tissue chemistry results were provided by animal technical staff who were blind to the goals of the study. Investigators performing analysis of samples for mass spectometry, RNA seq, and metabolomics were blinded to the allocation of mice. Investigators were not blinded for the in vitro experiments because the treatment groups were labeled, however all experiments were objective and conclusions based on multiple technical replicates and statistical significance.

Reporting for specific materials, systems and methods

We require information from authors about some types of materials, experimental systems and methods used in many studies. Here, indicate whether each material, system or method listed is relevant to your study. If you are not sure if a list item applies to your research, read the appropriate section before selecting a response.

Materials & experimental systems

n/a	Involved in the study
☐	☒ Antibodies
☐	☒ Eukaryotic cell lines
☒	☐ Palaeontology and archaeology
☐	☒ Animals and other organisms
☒	☐ Clinical data
☒	☐ Dual use research of concern

Methods

n/a	Involved in the study
☒	☐ ChIP-seq
☐	☒ Flow cytometry
☒	☐ MRI-based neuroimaging

Antibodies

Antibodies used	Anti-F4/80 (clone BM8); BioLegend; Catalog # 123106; Lot# B317518; 1:100 dilution<br>Rabbit anti rat IgG, mouse adsorbed; Vector Labs; Catalog # BA-4001; Lot ZE0201; 1:100 dilution
Validation	This product lot has passed BioLegend's QC testing and is certified for use.<br>Product citation: https://pubmed.ncbi.nlm.nih.gov/31167148/

Eukaryotic cell lines

Policy information about cell lines and Sex and Gender in Research

Cell line source(s)	The HepG2 liver hepatocellular carcinoma cell line was obtained from ATCC.
Authentication	The HepG2 cell line was not authenticated.
Mycoplasma contamination	All cell lines tested negative for mycoplasma contamination.
Commonly misidentified lines (See ICLAC register)	None used in this study

Animals and other research organisms

Policy information about studies involving animals; ARRIVE guidelines recommended for reporting animal research, and Sex and Gender in Research

Laboratory animals	Male C57Bl/6J mice (8-12 weeks of age) were used in this study. Dark/light cycle is 12/12 (6a-6p). Temperature 68-79F and humidity 30-70%.
Wild animals	No wild animals were used in the study
Reporting on sex	Male mice were used in this study to normalize for potential gender-related differences in animal activity, food intake, lipid storage and metabolism.
Field-collected samples	No field collected samples were used in the study
Ethics oversight	All mice were used in accordance with an approved NCI Frederick Animal Care and Use Committee protocol

Note that full information on the approval of the study protocol must also be provided in the manuscript.

Flow Cytometry

Plots

Confirm that:

☒ The axis labels state the marker and fluorochrome used (e.g. CD4-FITC).

☒ The axis scales are clearly visible. Include numbers along axes only for bottom left plot of group (a 'group' is an analysis of identical markers).

☒ All plots are contour plots with outliers or pseudocolor plots.

☒ A numerical value for number of cells or percentage (with statistics) is provided.

Methodology

Sample preparation	Liver tissues were homogenized in 200U/mg type 1 collagenase (Worthington Biochemical, Lakewood, NJ), 1mg/ml dispase 2 (Sigma) and 0.5 mg/ml DNase 1 (Sigma) using a GentleMacs tissue dissociator (Miltenyi Biotec). The single cell suspension was filtered through a 70 uM filter.

	Hepatocytes were isolated from freshly isolated mouse livers as described by Charni-Natan and Goldstein (STAR protocols 1, 100086, 2020; https://doi.org/10.1016/jxpro.2020.100086). Briefly, livers are perfused with warm saline followed by 25 ug/ml liberase. The digested livers are collected. Hepatocytes are released into suspension using sterile cell lifters and purified by differential centrifugation at 50 x g (2 min).
Instrument	BD Biosciences LSR-II
Software	BD FACS Diva Software for sample acquisition<br>FlowJo V10 software for sample analysis
Cell population abundance	Washed cells from livers were incubated with biotinylated anti-F4/80 antibody (clone BM8; BioLegend, San Diego, CA) followed by magnetically coupled streptavidin microbeads (Miltenyi Biotec). F4/80+ cells were positively selected by magnetic separation (Miltenyi BIotec) to >97% purity.<br><br>Hepatocytes from mouse livers were >95% pure based on visual inspection, noting cell morphology and size. Cell viability as confirmed by trypan blue exclusion.
Gating strategy	Viable cells were analyzed by plotting FSC/SSC such that low FSC cells are excluded. Doublet exclusion was performed by plotting the forward scatter height and areas. The geometric means of BODIPY staining were analyzed on all gated cells.

☒ Tick this box to confirm that a figure exemplifying the gating strategy is provided in the Supplementary Information.
